# LDH-mediated autophagic full-chain blockade for Multiple Myeloma treatment by targeting circ_0008255/miR-192-5p/ATG2A axis

**DOI:** 10.1016/j.bioactmat.2026.07.054

**Published:** 2026-08-04

**Authors:** Zhenhua Wang, Hefei Ren, Kun Wang, Xiaomin Zhang, Chang Liu, Hongkun Wu, Jieyun Shi, Jiafeng Zhang, Chaochao Wang, Liuzhi Wu, Juan Du, Fenyong Sun, Yelin Wu, Lin Zhou

**Affiliations:** a Department of Laboratory Medicine, Shanghai Changzheng Hospital, Naval Medical University, 415 Fengyang Road, Shanghai, 200003, China; b Institute of Hepatobiliary and Pancreatic Surgery, Department of Hepatobiliary and Pancreatic Surgery, Shanghai East Hospital, School of Medicine, Tongji University, Shanghai, 200120, China; c Department of Orthopedic Surgery, The Spine Surgical Center, Shanghai Changzheng Hospital, Naval Medical University, Shanghai, 200003, China; d Department of Hematology, Myeloma & Lymphoma Center, Shanghai Changzheng Hospital, Naval Medical University, Shanghai, 200003, China; e Department of Hematology, Renji Hospital, Shanghai Jiao Tong University School of Medicine, Shanghai. 200217, China; f Department of Clinical Laboratory Medicine, Tenth People's Hospital of Tongji University, Shanghai, 200072, China

**Keywords:** Autophagy, Full-chain autophagy blockade, Circ_0008255, Multiple myeloma, LDH

## Abstract

Multiple myeloma (MM) is an incurable plasma cell malignancy with limited therapeutic options. Although autophagy dysregulation is implicated in MM pathogenesis, its precise regulation, particularly by circular RNAs (circRNAs), is poorly understood. Through clinical RNA sequencing of primary MM patient samples, we identify an autophagy-associated circRNA, circ_0008255, which is markedly upregulated in MM patients and closely correlated with poor disease prognosis. Functional studies reveal that circ_0008255 promotes MM proliferation and tumor growth by enhancing autophagic activity. Mechanistically, it functions as a competitive endogenous RNA for miR-192-5p, leading to elevated expression of the core autophagy protein, autophagy related 2 homolog A (ATG2A). Furthermore, we developed a biomimetic nanoplatform based on layered double hydroxide (LDH) nanosheets coated with myeloma-derived cell membranes for tumor-specific delivery. This system co-delivers siRNA targeting circ_0008255 to suppress autophagosome initiation, while simultaneously leveraging the lysosome-alkalinizing property of LDH to impair autophagosome–lysosome fusion. Together, these actions enforce a synergistic autophagic full-chain blockade, leading to potent antitumor effects in vitro and in vivo. Overall, our study reveals a central regulatory role of circ_0008255 in myeloma autophagy, offering a promising therapeutic paradigm for MM.

## Highlights

• Circ_0008255 is upregulated in multiple myeloma and promotes tumor progression via the miR-192-5p/ATG2A/autophagy axis.
• A myeloma cell membrane-camouflaged LDH nanoplatform enables tumor-targeted delivery of circ_0008255 siRNA.
• This nanosystem realizes synergistic full-chain autophagy blockade and exhibits potent anti-myeloma efficacy in vitro and in vivo.

## Graphical abstract

Our study identified circ_0008255 as a significantly upregulated circRNA by RNA sequencing of primary multiple myeloma (MM) patient samples, and validated its clinical diagnostic potential by examining its expression in 121 MM patients and 80 healthy controls **(A)**.We further revealed a novel circ_0008255/miR-192-5p/ATG2A regulatory axis that drives autophagy, in which circ_0008255 acts as a molecular sponge for miR-192-5p to upregulate ATG2A, a core autophagy-related protein **(B)**.Finally, we constructed an MM cell membrane-coated LDH nanosheets loaded with circ_0008255 siRNA (denoted LDH@siRNA@MM, LSM).This platform integrates the siRNA-stabilizing and autophagy-inhibiting properties of LDH with MM membrane-mediated tumor targeting, enabling circ_0008255 silencing to block autophagosome initiation and LDH-induced lysosomal alkalinization to suppress autophagosome–lysosome fusion, thus achieving full-chain autophagy blockade for effective MM therapy **(C)**.

**Figure:**
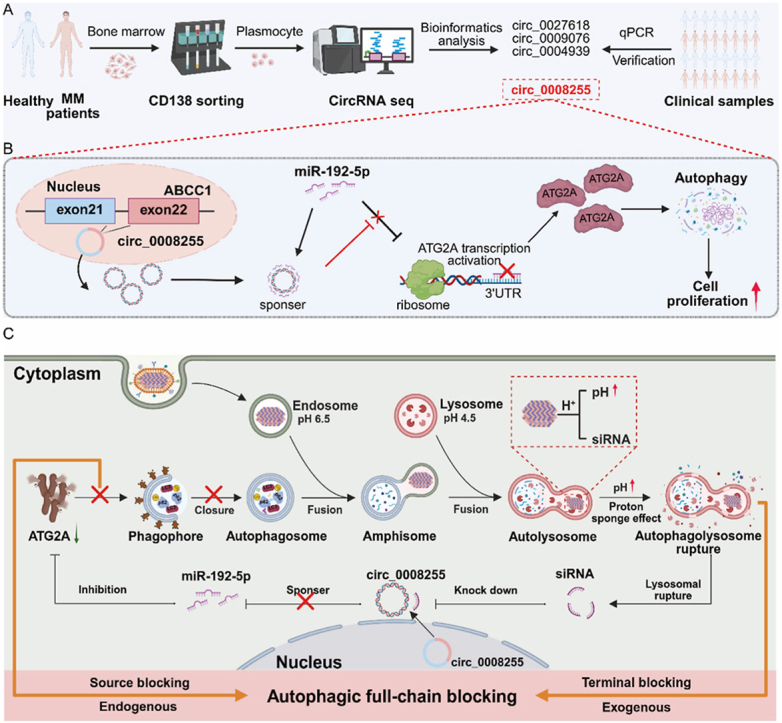

## 1 Introduction

Multiple myeloma (MM), which accounts for nearly 1% of all cancers and approximately 13% of all hematologic malignancies, is a plasma cell malignancy characterized by the presence of abnormal clonal plasma cells in bone marrow and typically accompanied by the secretion of monoclonal immunoglobulins, bone-destructive lesions, renal impairment, anemia, and hypercalcemia [1,2]. Despite emerging novel interventions such as proteasome inhibitors, immune modulators, and biological therapies that have improved the outcome of MM patients, the survival rate of MM remains poor [3]. Hence, uncovering the molecular mechanisms of MM pathogenesis and developing novel therapeutic strategies hold profound significance for MM patients.

CircRNAs are important members of the non-coding RNA molecules which are characterized by the covalently closed loop structures and the absence of 3′ and 5’ ends, and they are also implicated in tumorigenesis and progression [4]. An increasing number of studies reported that some circRNAs play key roles in tumor progression and metastasis through diverse mechanisms, including miRNA sponges, RNA-binding protein (RBP) interactions, gene transcription and translation regulators [5]. Accordingly, circRNAs may be one of the promising biomarkers and therapeutic targets for different carcinomas [6]. Recently, altered circRNA expression has been implicated in the tumorigenesis of MM, likely through a sponge mechanism [7]. However, MM-associated circRNAs as well as their effects on regulating autophagy and MM progression are little known.

Autophagy is a conserved biological process through which cellular components and damaged organelles are sequestered in autophagosomes for lysosomal degradation, and it plays dual roles by promoting both death and survival of cancer cells [8]. As a pivotal mechanism sustaining tumor cell survival, the autophagy pathway has emerged as a prominent potential target in cancer therapy [9]. However, the repertoire of identified autophagy modulators remains limited. Most research focuses on blocking the lysosomal degradation phase of autophagy, utilizing agents such as hydroxychloroquine (HCQ) and the lysosomal autophagy inhibitor Lys05 [10]. Additionally, certain studies employ inhibitors of UNC51-like kinase 1 (ULK1) or VPS34, which act on the initiation phase of autophagy [11,12]. All the aforementioned inhibitors target solely a single stage in the autophagic process, thereby necessitating high concentrations to exert their effects. This not only tends to elicit a range of adverse reactions but also severely constrains the clinical application of autophagy-targeted therapies in oncology. Consequently, the construction of a multi-target synergistic autophagy-blocking system to enhance autophagy inhibition efficiency holds substantial significance for improving tumor therapeutic efficacy.

**Fig. 1:**
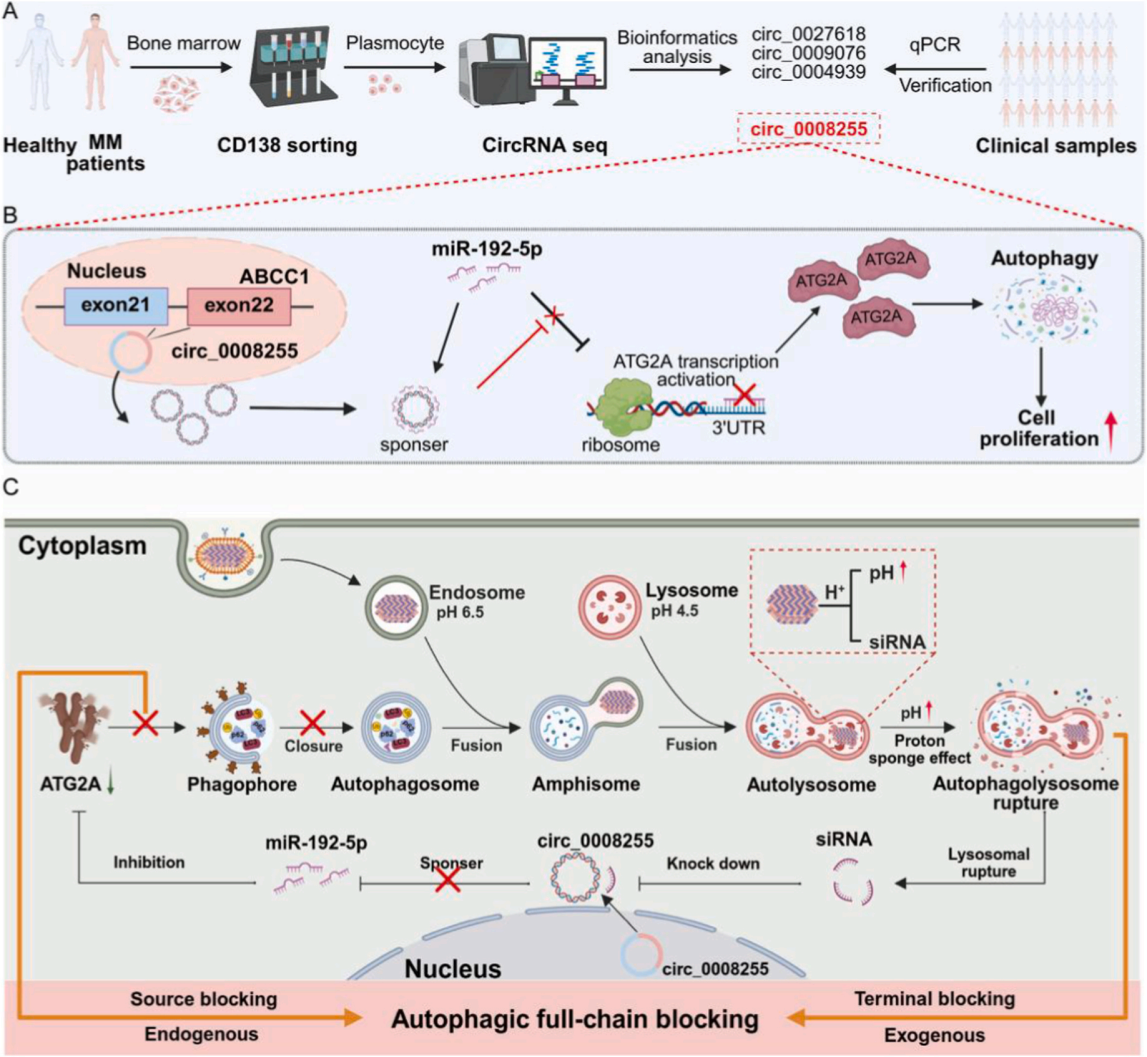
From screening to target: a schematic diagram of circ_0008255's diagnostic value and mechanisms in multiple myeloma. **A)** Schematic diagram of the circ_0008255 screening process. **B)** Schematic diagram of circ_0008255 promoting malignant progression in multiple myeloma. **C)** Schematic diagram of LSM nanosheets inhibiting malignant progression of MM via the Autophagic full-chain Blocking. Panel Fig. 1 partially created with BioRender.

In this study, we systematically investigate the role of a novel circRNA (circ_0008255) in MM progression and autophagy regulation. As shown in Fig. 1A, we first identified circ_0008255 as a highly expressed circRNA via RNA sequencing of primary MM patient samples. Then we conformed its clinical diagnostic utility by detecting the expression of circ_0008255 from 121 MM patients and 80 healthy controls. For functional characterization, gain-of-function and loss-of-function assays demonstrated that circ_0008255 significantly promotes MM cell proliferation both in vitro and in vivo. Mechanistically, we unravel a circ_0008255/miR-192-5p/ATG2A axis that promotes autophagy, where circ_0008255 sponges miR-192-5p to upregulate ATG2A, a critical autophagy-related protein (Fig. 1B). Small interfering RNA (siRNA) offers distinct advantages for disease treatment. It achieves precise gene silencing through complementary base pairing and can target hard-to-drug non-coding RNAs that small-molecule drugs fail to act on [13]. To target this axis, we engineer an MM membrane-coated LDH nanosheets loaded with circ_0008255 siRNA (LDH@siRNA@MM, LSM). As shown in Fig. 1C, this platform leverages LDH's siRNA-stabilizing capacity and autophagy-inhibiting properties, combined with MM membrane-mediated targeting, to silence circ_0008255 and thereby block autophagosome initiation and LDH-induced lysosomal alkalinization to inhibit autophagosome-lysosome fusion, thereby achieving a full-chain autophagic blocking for MM treatment.

Our findings not only reveal a novel circRNA-driven autophagy regulatory network in MM but also present an LDH-based efficient autophagy-blocking, bridging basic mechanistic insights with translational applications. This work underscores the potential of circRNAs as both biomarkers and therapeutic targets, while offering a paradigm for designing targeted delivery systems to improve RNAi-based cancer therapies.

## 2 Results and discussion

### 2.1 RNA-seq profiling reveals a novel overexpressed circRNA in MM

**Fig. 2:**
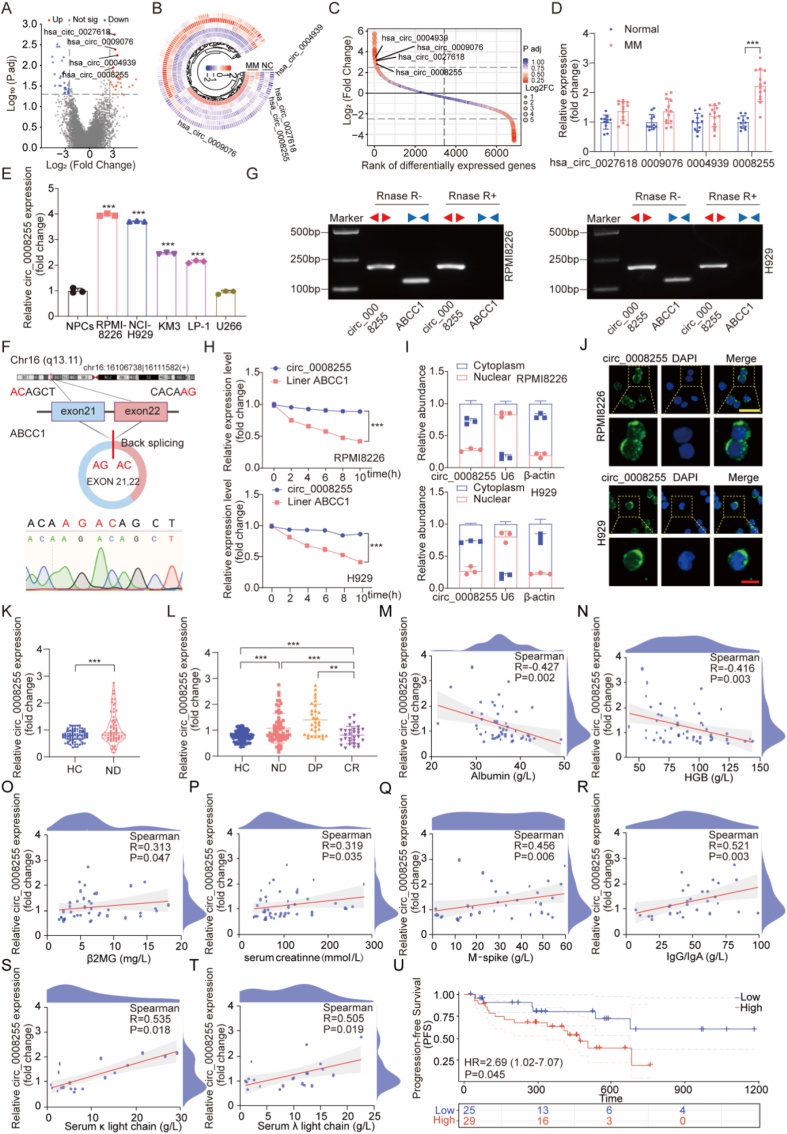
**RNA-seq profiling identifies a novel overexpressed circRNA that is closely associated with poor prognosis in MM**. **A**) Collection of bone marrow samples from 3 healthy controls and 3 newly diagnosed MM patients for high-throughput RNA-seq. Differential genes identified based on the volcano plot**)** with the top 4 significantly upregulated circRNAs labeled. **B)** Heatmap representation of RNA-seq results. **C)** Differential expression plot of RNA-seq data. **D)** Measurement of the expression levels of the 4 circRNAs in bone marrow samples from 11 healthy controls and 14 newly diagnosed MM patients. **E)** Detection of circ_0008255 expression in normal plasma cells and MM cell lines. **F)** RT-qPCR and Sanger sequencing confirmation of the circularization site of circ_0008255 using RPMI8226 cell RNA as a template. **G)** Total RNA (2 μg) was treated with 2 U/μg of RNase R at 37°C. Normal PCR detection of ABCC1 and circ_0008255 after RNase R treatment of RPMI8226 and NCI-H929 cell RNA. **H)** RT-qPCR analysis of RPMI8226 and NCI-H929 expression following actinomycin D treatment. **I)** Measurement of circ_0008255 expression differences in the cell nucleus and cytoplasm using nuclear-cytoplasmic fractionation. **J)** FISH to observe the cellular localization of circ_0008255 (Yellow scale bar = 25 μm,Red scale bar = 100 μm). **K)** Expression levels of circ_0008255 in peripheral blood plasma of 61 newly diagnosed MM patients and 80 healthy controls. **L)** Expression levels of circ_0008255 in peripheral blood plasma of 80 healthy controls and MM patients at different disease stages (61 newly diagnosed MM**)** 30 disease progressive MM**)** and 30 complete remission stage of MM). **M)** Correlation of circ_0008255 expression levels with albumin (ALB). **N)** Correlation of circ_0008255 expression levels with hemoglobin (HGB). **O)** Correlation of circ_0008255 expression levels with β2-microglobulin (β2-MG). **P)** Correlation of circ_0008255 expression levels with creatinine (Cr). **Q)** Correlation of circ_0008255 expression levels with M protein. **R)** Correlation of circ_0008255 expression levels with immunoglobulin (IgG or IgA). **S)** Correlation of circ_0008255 expression levels with kappa light chain. **T)** Correlation of circ_0008255 expression levels with lambda light chain. **U)** Kaplan-Meier curve depicting progression-free survival in MM patients with high expression of circ_0008255. n.s. *P > *0.05, *P < 0.05, **P < 0.01, ***P < 0.001.

To identify circRNAs that might contribute to MM pathogenesis, we performed high-throughput RNA sequencing (RNA-seq) of bone marrow samples from 3 pairs of newly diagnosed MM patients and healthy donors. The results showed that 1273 circRNAs were significantly altered with |log2 fold-change| >1.0 and P value < 0.05. Among them, 314 circRNAs have been reported in circBase, including 189 upregulated and 125 downregulated circRNAs (Fig. S1A–C). With |log2 fold-change| > 2.9 and P value < 0.05 as the threshold criteria, we found 4 circRNAs, hsa_circ_0004939, hsa_circ_0027618, hsa_circ_0008255, and hsa_circ_0009076, as the most differentially expressed circRNAs in the upregulated group (Fig. 2A–C). Reverse transcription-quantitative polymerase chain reaction (RT-qPCR) analysis of bone marrow samples from 14 newly diagnosed MM patients and 11 healthy adults indicated that among these 4 circRNAs, hsa_circ_0008255 (circ_0008255), a novel circRNA without any identified function, was most significantly overexpressed in MM patients (Fig. 2D). We then assessed circ_0008255 expression in 5 MM cell lines (U266, NCI-H929, RPMI8226, LP-1 and KM3) and normal plasma cells (NPCs). As shown in Fig. 2E, circ_0008255 was also significantly overexpressed in 4 out of 5 MM cell lines (RPMI8226, NCI-H929, KM-3 and LP-1) as compared to normal PCs, with RPMI8226 and NCI-H929 cells exhibiting the highest expression levels.

CircRNAs are characterized by a covalently closed loop structure that is generated by back-splicing, eventually leading to increased structural stability [14]. To investigate these properties of circ_0008255, Sanger sequencing and RNase R treatment were used in RPMI8226 and NCI-H929 cells. Sanger sequencing confirmed the head-to-tail splicing of the amplified circ_0008255, suggesting that circ_0008255 was derived from exons 21 and 22 of ATP binding cassette sub-family C member 1 (ABCC1) gene, consistent with the circ_0008255 data from CircBase (Fig. 2F). We then extracted RNAs from RPMI8226 and NCI-H929 MM cells respectively, and treated these RNAs with or without RNase R (a degrader of the linear RNA), generated cDNA from the cells, and subsequently examined whether circ_0008255 and linear ABCC1 could be detected from the cDNA by RT-qPCR analysis. Results showed that circ_0008255 was detected in the cDNA using divergent primers, even under RNase R treatment (Fig. 2G). As a control, linear ABCC1 transcripts were detected only in the absence of RNase R treatment (Fig. 2G). Therefore, circ_0008255 indeed exists in a circular form. Furthermore, we evaluated the stability of circ_0008255 by using actinomycin D to prevent new transcription, thus allowing the chasing of already-transcribed RNA. The results showed that the half-life of circ_0008255 was longer than that of linear ABCC1, which suggested that circ_0008255 was more stable in MM cells (Fig. 2H). Next, to explore the subcellular localization of circ_0008255, we performed nuclear and cytoplasmic extraction assays in MM cells, and RT-qPCR analysis showed that circ_0008255 was mostly expressed in the cytoplasm rather than in the nucleus (Fig. 2I). Fluorescence in situ hybridization (FISH) assays also revealed that circ_0008255 was primarily located in the cytoplasm (Fig. 2J). Collectively, these data suggest that circ_0008255 is a stable and highly expressed circRNA in MM.

### 2.2 Heightened expression of circ_0008255 is closely associated with the poor prognosis of MM

To further corroborate the clinical relevance of circ_0008255 in MM progression using a minimally invasive approach, we first examined circ_0008255 expression levels in peripheral blood plasma samples of 61 newly diagnosed MM patients and 80 healthy donors. The clinical and pathological characteristics of 61 individuals newly diagnosed with MM were summarized in Table S1 and Table S2. The results showed that the expression level of circ_0008255 was significantly increased in MM primary samples than those from healthy donors (Fig. 2K). Notably, we also found that circ_0008255 expression was significantly elevated in both newly diagnosed (n = 61) and disease progressing (n = 30) patients compared to healthy donors (n = 80) and MM patients in the stage of complete remission (n = 30), with levels being higher in progressive than newly diagnosed patients (Fig. 2L).

Then we investigated the relationship between circ_0008255 expression levels and the clinicopathological characteristics of newly diagnosed MM patients. As shown in Fig. 2M–T, circ_0008255 expression levels negatively correlated with levels of albumin (ALB) (Fig. 2M) and hemoglobin (HGB) (Fig. 2N), whereas positively correlated with levels of serum β2-microglobulin (β2-MG) (Fig. 2O), creatinine (Cr) (Fig. 2P), M protein (Fig. 2Q), immunoglobulin (IgG or IgA) (Fig. 2R), kappa light chain (Fig. 2S), and lambda light chain (Fig. 2T), respectively, which are all hallmarks of tumor mass and disease activity in myeloma. Furthermore, we analyzed the Kaplan-Meier progression-free survival (PFS) of the MM patients we followed up previously. The survival data showed that patients with high levels of circ_0008255 had obviously shorter progression-free survival (Fig. 2U).

In addition, Receiver-operating characteristic (ROC) curves were plotted for the MM group and control group to assess the diagnostic potential of circ_0008255 in MM patients. The sensitivity and specificity of circ_0008255 were verified by this method, and the AUC value was calculated as 0.698 (95% CI: 0.605-0.790) (Table S3). In the combined ROC analysis, the logistic regression model using circ_0008255, ALB and HB resulted in a higher AUC of 0.964 (95% CI: 0.93-0.998) and higher performance (sensitivity 89.7%, specificity 97.4%) compared with the only albumin or hemoglobin (Fig. S1D–F). Overall, these findings indicate that circ_0008255 overexpression is closely correlated with MM progression and thus it might be potentially used as a diagnostic and prognostic biomarker for MM.

### 2.3 Circ_0008255 aggravates the malignant properties of MM cells in vitro and in vivo

**Fig. 3:**
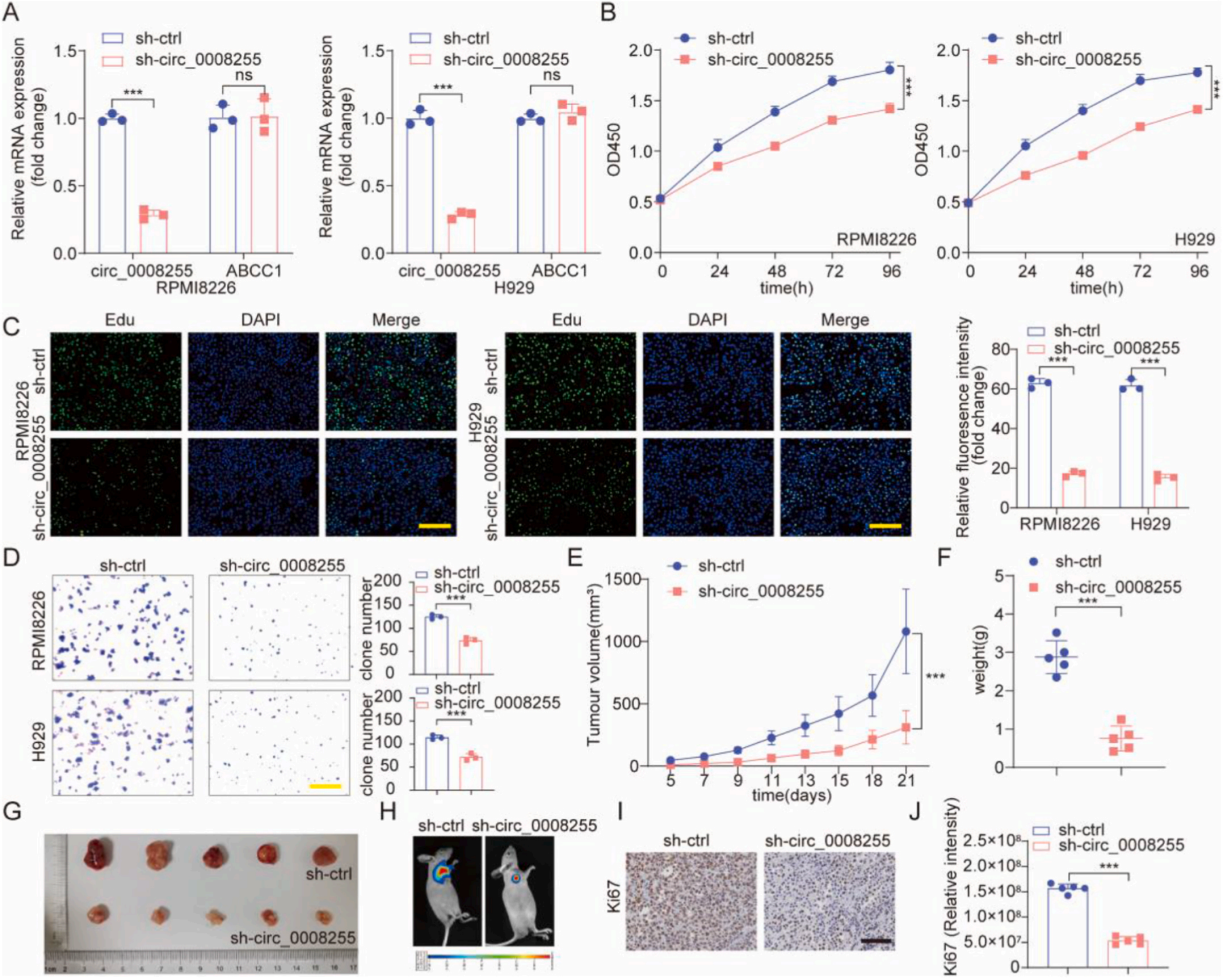
**Circ_0008255 aggravates the malignant properties of MM cells in vitro and in vivo**. **A)** Detection of circ_0008255 and ABCC1 after knocking down circ_0008255 by RT-qPCR. **B)** Cell proliferation changes in RPMI8226 and H929 cells infected with sh-ctrl and sh-circ_0008255 viruses measured by CCK-8 assay. **C)** Cell proliferation changes in RPMI8226 and H929 cells infected with sh-ctrl and sh-circ_0008255 viruses assessed by EdU staining (Scale bar = 500 μm). **D)** Colony formation assay evaluating cell proliferation changes in RPMI8226 and H929 cells infected with sh-ctrl and sh-circ_0008255 viruses (Scale bar = 500 μm). **E)** Tumor volume measured at different time points after subcutaneous injection of RPMI8226 cells overexpressing luciferase and infected with sh-ctrl and sh-circ_0008255 viruses in nude mice. **F)** Tumor weight measurement. **G)** Images of tumor size on day 21. **H)***In vivo* imaging of tumor-bearing mice. **I, J)** Immunohistochemical detection of Ki67 expression in tumors (Scale bar = 100 μm). All the data are presented as the mean ± standard deviation from 3 independent experiments, n.s. *P > *0.05, *P < 0.05, **P < 0.01, ***P < 0.001.

To explore the biological functions of circ_0008255 in MM, circ_0008255 was knocked down by circ_0008255-targeting shRNA (sh-circ_0008255), and RT-qPCR was performed to verify the knockdown efficiency of circ_0008255 and mRNA levels of ABCC1 in both RPMI8226 and NCI-H929 cells (Fig. 3A). Western blot was performed to detect the protein expression level of ABCC1 after circ_0008255 knockdown (Fig. S2A). These results revealed that sh-circ_0008255 markedly suppressed the mRNA expression of circ_0008255, while exerting no effects on both the mRNA and protein levels of ABCC1. We first examined the effect of circ_0008255 on cell proliferation, and CCK-8 assay showed knockdown of circ_0008255 impaired the proliferation of MM cells (Fig. 3B). Consistently, 5-ethynyl-2′-deoxyuridine (EdU) assay also revealed that circ_0008255 knockdown significantly inhibited cell proliferation in both RPMI8226 and NCI-H929 cells (Fig. 3C). Moreover, in a clonogenic soft agar assay, knockdown of circ_0008255 diminished clonal formation in MM cells (Fig. 3D).

Subsequently, we employed a MM cell-derived xenograft mouse model to investigate whether circ_0008255 could influence tumor growth in vivo. As shown in Fig. 3E–H, RPMI8226 cells with stable knockdown of circ_0008255 significantly reduced tumor growth, as indicated by tumor size and weight (Fig. 3E–H, Fig. S2B). Immunohistochemical staining of Ki-67 in the tumors transduced with sh-circ_0008255 also showed that circ_0008255 knockdown markedly attenuated cell proliferation in vivo (Fig. 3I and J). Altogether, these results demonstrate that circ_0008255 promote MM cellular proliferation, and tumor growth both in vitro and in vivo.

### 2.4 Circ_0008255 promotes autophagy in MM cells

**Fig. 4:**
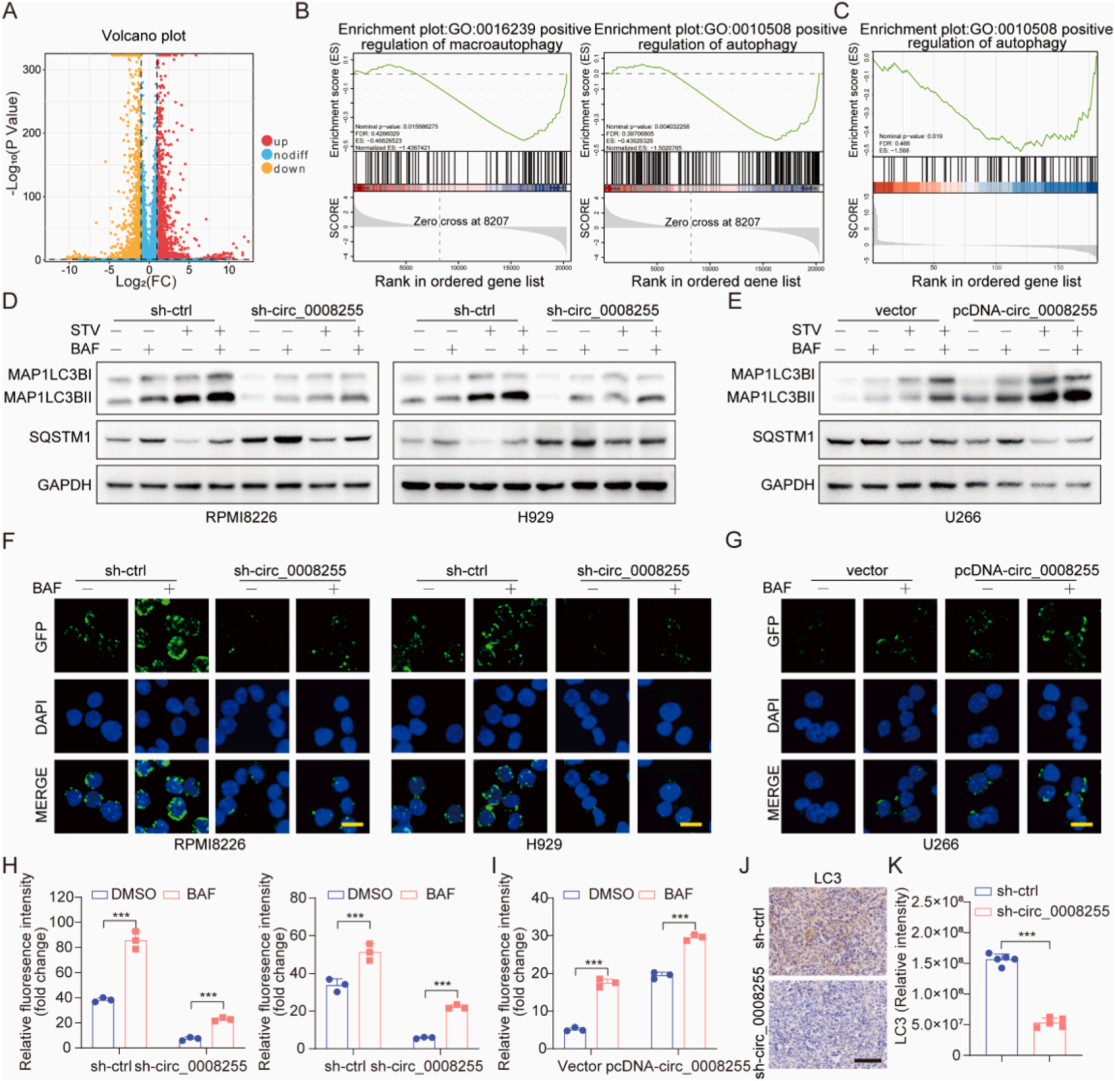
**Circ_0008255 promotes autophagy in MM cells**. **A**) Collection of RPMI8226 cells infected with sh-ctrl and sh-circ_0008255 viruses, for high-throughput RNA-seq. Differential genes identified based on the volcano plot with *P* < 0.05, |log2FC|>2. **B)** GSEA showing enrichment of autophagy signaling pathways in knockdown of circ_0008255 and control group. **C)** GSEA analysis based on expression levels of autophagy-related genes in normal and MM patients. **D)** RPMI8226 and H929 cells were infected with sh-ctrl and sh-circ_0008255 viruses, and then incubated with normal or starvation medium Earle's balanced salt solution (EBSS) as indicated for 2 h, in the absence or presence of 20 nM BAF. Whole-cell extracts were subjected to immunoblotting with anti-LC3B and SQSTM1 antibodies. GAPDH was used as loading control. **E)** U266 cells were infected with vector and pcDNA-circ_0008255 viruses, and then incubated with normal or starvation medium EBSS as indicated for 2 h, in the absence or presence of 20 nM BAF. Whole-cell extracts were subjected to immunoblotting with anti-LC3B and SQSTM1 antibodies. GAPDH was used as loading control. **F, H)** RPMI8226 and H929 cells stably expressing GFP-LC3B infected with sh-ctrl and sh-circ_0008255 viruses, stimulated with BAF (20 nM) for 4 h. Observation of cell autophagy using confocal microscopy. Representative images are shown (Scale bar = 25 μm). **G, I)** U266 cells expressing GFP-LC3B infected with vector and pcDNA-circ_0008255 viruses, stimulated with BAF (20 nM) for 4 h. Observation of cell autophagy using confocal microscopy (Scale bar = 25 μm). **J, K)** Immunohistochemical detection of LC3B expression in tumors (Scale bar = 100 μm). Representative images are shown. All the data are presented as the mean ± standard deviation from 3 independent experiments, n.s. *P > *0.05, *P < 0.05, **P < 0.01, ***P < 0.001.

To further investigate the mechanism by which circ_0008255 affects MM cellular proliferation and tumor growth, we performed RNA sequencing in circ_0008255 silencing MM cells and identified several differentially expressed genes (Fig. 4A). Gene Set Enrichment Analysis (GSEA) revealed a significant enrichment of the autophagy signaling pathway in these genes (Fig. 4B). Additionally, we performed GSEA analysis on differentially expressed autophagy genes between normal controls and MM patients from Gene Expression Omnibus (GEO) (https://www.ncbi.nlm.nih.gov/geo/) and found a similar enrichment of the autophagy signaling pathway (Fig. 4C, Fig. S3A). We therefore sought to examine the effect of circ_0008255 on autophagy in MM cells.

The conversion of the soluble form of light chain 3B (LC3B-I) to a lipidated and autophagosome-associated form (LC3B-II) is a hallmark of autophagy, and SQSTM1 (sequestosome1)/SQSTM1 is an autophagic receptor accumulated in cells when autophagy flux is suppressed [15]. We first performed Western blot analysis to check these autophagic markers in MM cells upon circ_0008255 knockdown or overexpression. The results showed that under both basal and starvation conditions, suppression of circ_0008255 reduced LC3B-II conversion and SQSTM1 degradation in both RPMI8226 and NCI-H929 cells (Fig. S3B and Fig. 4D) while overexpression of circ_0008255 caused an opposite effect in U266 cells, which expressed a lower level of endogenous circ_0008255 (Fig. 4E). Furthermore, the immunofluorescence images showed that circ_0008255 knockdown decreased formation of GFP-LC3B puncta (Fig. 4F–H), whereas its overexpression increased GFP-LC3B puncta formation (Fig. 4G–I).

Next, we used the lysosomal V-ATPase inhibitor bafilomycin A1 (BAF), a late-phase autophagy inhibitor that prevents autophagosome-lysosome fusion and LC3B-II degradation, and found that the effect of BAF on LC3B-II accumulation was reduced by circ_0008255 depletion (Fig. 4E, F and H), whereas circ_0008255 overexpression further increased LC3B-II accumulation upon BAF treatment (Fig. 4E–G and I). Additionally, we treated MM cells with autophagy inhibitors, 3-methyladenine (3-MA), and autophagy inducers (rapamycin) followed by circ_0008255 depletion, and found that suppression of circ_0008255 attenuated the accumulation of LC3B-II in 3-MA and rapamycin-treated cells (Fig. S3C–D). Eventually, we confirmed that circ_0008255 knockdown markedly attenuated cell autophagy in vivo by immunohistochemical staining of LC3B-II (Fig. 4Jand K). Taken together, our data reveal that circ_0008255 silencing inhibits autophagic flux in MM cells.

### 2.5 Circ_0008255 functions as a miR-192-5p sponge to regulate autophagy in MM cells

**Fig. 5:**
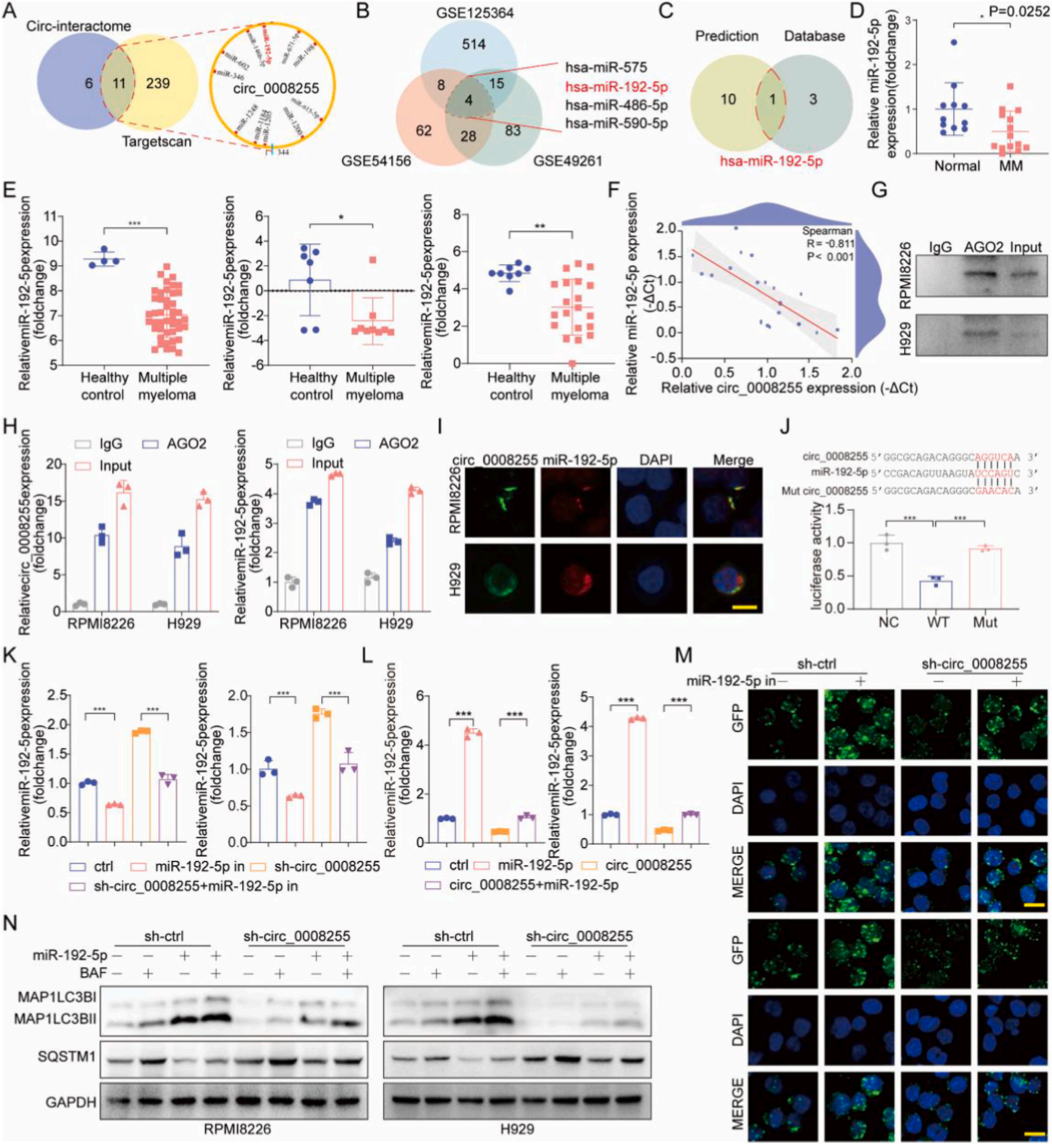
**Circ_0008255 functions as a miR-192-5p sponge to regulate autophagy in MM cells**. **A**) Prediction of miRNAs potentially targeting circ_0008255 using circ-interactome and targetscan websites, and Venn diagram intersection analysis. **B)** Analysis of downregulated miRNAs in GSE125364, GSE54156, and GSE49261 databases, and Venn diagram intersection analysis. **C)** Venn diagram intersection analysis of results from Fig. A and Fig. B. **D)** Expression levels of miR-192-5p detected by RT-qPCR in peripheral blood plasma from 11 normal individuals and 14 MM patients. **E)** Differential expression of miR-192-5p in normal individuals and MM patients in GSE125364, GSE54156, and GSE49261 databases. **F)** Analysis of expression correlation between circ_0008255 and miR-192-5p in 19 MM patients. **G, H)** RIP experiment using AGO2 antibody to detect AGO2 protein, and RT-qPCR detection of circ_0008255 and miR-192-5p expression. **I)** FISH staining for cellular localization of circ_0008255 (green) and miR-192-5p (red) (Scale bar = 20 μm). **J)** Luciferase reporter gene assay confirming miR-192-5p can target circ_0008255. **K)** RT-qPCR detection of miR-192-5p expression in MM cells with simultaneous or separate knockdown of circ_0008255 and miR-192-5p. **L)** RT-qPCR detection of miR-192-5p expression in MM cells with simultaneous or separate overexpression of circ_0008255 and miR-192-5p. **M)** Confocal microscopy observation of cell autophagy in RPMI8226 and H929 cells stably expressing GFP-LC3B with simultaneous or separate knockdown of circ_0008255 and miR-192-5p (Scale bar = 25 μm). **N)** Western blot detection of LC3B and SQSTM1 expression in RPMI8226 and H929 cells stably expressing GFP-LC3B with simultaneous or separate knockdown of circ_0008255 and miR-192-5p after BAF (20 nM) stimulation for 4 h. All the data are presented as the mean ± standard deviation from 3 independent experiments, n.s. *P > *0.05, *P < 0.05, **P < 0.01, ***P < 0.001.

CircRNAs can exert their functions through diverse mechanisms, including microRNA (miRNA) sponging, protein translation, direct protein binding, and influencing stability [6]. Specifically, circRNAs distributed in the cytoplasm can bind to certain miRNAs by acting as miRNA sponges to regulate gene transcription and translation [6]. Owing to the distribution of circ_0008255 in the cytoplasm of MM cells (Fig. 2I and J), we hypothesized that circ_0008255 might sponge miRNA to regulate autophagy in MM cells. To identify miRNAs which can bind to circ_0008255, two public prediction tools, circ-interactome (https://circinteractome.irp.nia.nih.gov/) and Target Scan (https://www.targetscan.org/vert_80/) databases, were used. A Venn diagram was constructed, showing 11 potential target miRNAs (Fig. 5A). Subsequently, we searched for miRNAs with downregulation profiling in human MM using three public GEO databases and identified 4 miRNA candidates (Fig. 5B). Based on these two predicted results, miR-192-5p was identified as a candidate miRNA sponge for circ_0008255 (Fig. 5C).

Subsequently, we found that the expression levels of miR-192-5p were markedly lower in MM peripheral blood plasma samples than those in healthy donors (Fig. 5D), and the downregulation of miR-192-5p was also confirmed in MM patient samples from the GEO database (Fig. 5E). Further analysis indicated that miR-192-5p expression levels were inversely associated with circ_0008255 expression in MM primary samples (Fig. 5F). Next, we performed RNA immunoprecipitation (RIP) with argonaute 2 (AGO2) antibody in RPMI8226 and NCI-H929 cells. As expected, our results showed that both circ_0008255 and miR-192-5p were efficiently precipitated by the AGO2 antibody (Fig. 5G and H). Biotin-based RNA pull-down further validated their physical interaction. Compared with scrambled probe control, the specific circ_0008255 probe dramatically enriched endogenous miR-192-5p (Fig. S4A), which together with AGO2 RIP results confirms the direct binding of circ_0008255 to miR-192-5p. In addition, FISH analysis in RPMI8226, NCI-H929 and U266 cells revealed that miR-192-5p was co-localized with circ_0008255 in the cytoplasm, suggesting an interaction between circ_0008255 and miR-192-5p (Fig. 5I, Fig. S4B). To further verify the direct binding between circ_0008255 and miR-192-5p, we performed a dual-luciferase reporter assay in 293T cells. As shown in Fig. 5J, miR-192-5p significantly inhibited the luciferase reporter activity of the wild type but not the miR-192-5p-target mutant circ_0008255, indicating that miR-192-5p was a direct target of circ_0008255. Lastly, RT-qPCR results showed that knockdown of circ_0008255 significantly increased miR-192-5p expression in MM cells, while treatment with synthetic miR-192-5p inhibitor attenuated the effect of circ_0008255 on the expression of miR-192-5p (Fig. 5K). However, the overexpression of circ_0008255 significantly inhibited miR-192-5p expression in MM cells, while treatment with synthetic miR-192-5p mimics attenuated the effect of circ_0008255 on the expression of miR-192-5p (Fig. 5L).

Previous report has described that miR-192-5p was downregulated in human MM and associated with disease progression [16]. By conducting a series of experiments, including CCK-8, EdU, and colony formation assays, we demonstrated that forced expression of miR-192-5p significantly inhibited the proliferation of MM cells (Fig. S5A–E), suggesting that miR-192-5p inhibits MM progression. Next, we investigated whether miR-192-5p could affect autophagy levels in MM cells. Western blotting showed decreased LC3B-II level and increased SQSTM1 level in RPMI8226 and NCI-H929 cells after transfection with miR-192-5p mimics (Fig. S5F). Conversely, LC3B-II was increased, and SQSTM1 was decreased after synthetic miR-192-5p inhibitor transfection (Fig. S5G). Moreover, immune-fluorescence analysis showed LC3B dots were decreased in the MM cells transfected with miR-192-5p mimics (Fig. S5H) but increased in those transfected with synthetic miR-192-5p inhibitor, indicating that miR-192-5p inhibits autophagy in MM cells.

Last, we directly tested whether circ_0008255 regulates autophagy of MM cells via sponging miR-192-5p. In RPMI8226 and NCI-H929 cells, synthetic miR-192-5p inhibitor abolished the effect of circ_0008255 knockdown on autophagy as measured by LC3B-II level (Fig. 5M and Fig. S6). Conversely, knockdown of circ_0008255 attenuated the autophagy-stimulating effect of synthetic miR-192-5p inhibitor, either with or without BAF treatment (Fig. 5N). In conclusion, these data confirm that circ_0008255 regulates autophagy by sponging miR-192-5p.

### 2.6 ATG2A is the downstream target of circ_0008255/miR-192-5p axis and exerts oncogenic effects by modulating autophagy in MM

**Fig. 6:**
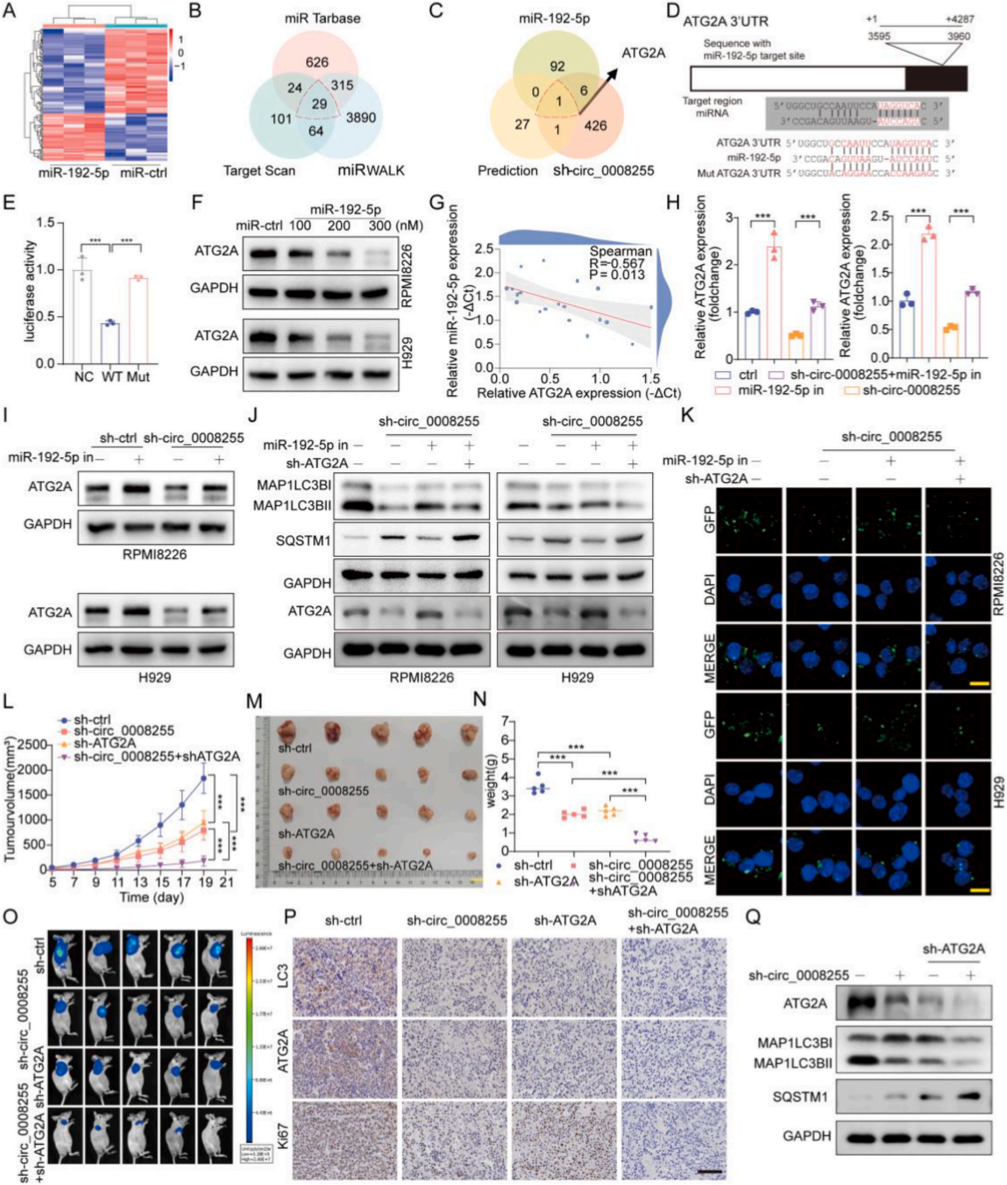
**ATG2A is the downstream target of circ_0008255/miR-192-5p axis and exerts oncogenic effects in MM by modulating autophagy**. **A)** Heatmap analysis based on RNA-seq results of overexpression of miR-192-5p in RPMI8226 cells. **B)** Three bioinformatics databases miR Walk, miR Tarbase, and Target Scan were utilized to predict the target genes of miR-192-5p. **C)** Venn diagram analysis comparing the RNA-seq results with website analysis. **D)** Binding sites and mutation sites of miR-192-5p on the 3′UTR of ATG2A. **E)** Luciferase reporter gene assay. **F)** Detection of ATG2A protein level changes upon overexpression of miR-192-5p in MM cells. **G)** Analysis of expression correlation between miR-192-5p and ATG2A in 19 MM patients. **H)** Detection of ATG2A mRNA level changes upon simultaneous or separate knockdown of circ_0008255 and miR-192-5p in MM cells. **I)** Detection of ATG2A protein level changes upon simultaneous or separate knockdown of circ_0008255 and miR-192-5p in MM cells. **J)** MM cells divided into 4 groups: control group, circ_0008255 knockdown group, circ_0008255 knockdown + miR-192-5p inhibitor group, circ_0008255 knockdown + miR-192-5p inhibitor + ATG2A knockdown group, followed by detection of ATG2A, LC3B and SQSTM1 expression. **K)** MM cells stably overexpressing GFP-LC3B divided into 4 groups: control group, circ_0008255 knockdown group, circ_0008255 knockdown + miR-192-5p inhibitor group, circ_0008255 knockdown + miR-192-5p inhibitor + ATG2A knockdown group, subjected to confocal microscopy for autophagic flux detection (Scale bar = 25 μm). **L)** Tumors were measured with an electronic caliper on days 5/7/9/11/13/15/17/19 in nude mice followed by statistical analysis. Averaged tumor volume of each group mean ± SD shown. **M)** The excised tumor from all the mice in each group. Comparison of tumor volume on day 19 after tumor removal. **N)** Weighing analysis of the tumors. Data were presented as the mean ± SD from five mice of each group. **O)** Live imaging of nude mice on day 19. P**)** IHC detection of LC3B, ATG2A, and ki-67 expression in the tumors (Scale bar = 100 μm). **Q)** Western blot detection of ATG2A, LC3B and SQSTM1 expression in the tumors. All the data are presented as the mean ± standard deviation from 3 independent experiments, n.s. *P > *0.05, *P < 0.05, **P < 0.01, ***P < 0.001.

To further explore the direct downstream target gene of miR-192-5p, we investigated mRNAs that might be regulated by miR-192-5p in MM cells via RNA sequencing of MM cells transfected with miR-192-5p mimics. The initial screening identified 99 down-regulated mRNAs in RPMI8226 cells with miR-192-5p overexpression (P value < 0.05 and |log2 fold-change| >1) (Fig. 6A, Fig. S8A). Three bioinformatics databases, miR Walk (https://ngdc.cncb.ac.cn/database commons/database/id/3941), miR Tarbase (http://mirtarbase.mbc.nctu.edu.tw/php/-index.php), and Target Scan, were also used to predict miR-192-5p target gene and obtained 29 potential target genes (Fig. 6B). We then performed Venn analysis of these two results (Fig. 6A and B and genes down-regulated in circ_0008255-depleted cells obtained from our previous RNA-seq data (Fig. 4A), and eventually identified a single overlapped gene, the autophagy-related gene ATG2A (Fig. 6C), with a predicted interaction between miR-192-5p and its 3ʹ-untranslated region (3ʹ-UTR) (Fig. 6D).

Consistent with our bioinformatic analysis, transfection of miR-192-5p mimics reduced the luciferase activity of the wild-type ATG2A 3ʹ-UTR reporter, but not the one with miR-192-5p binding site mutated (Fig. 6E), indicating that ATG2A is a direct target gene of miR-192-5p. This finding was further confirmed by the significantly decreased expression of ATG2A at both the mRNA (Fig. S8B) and protein levels (Fig. 6F) in MM cells following the ectopic expression of miR-192-5p. Moreover, the levels of the ATG2A were negatively correlated with miR-192-5p levels in MM primary samples (Fig. 6G). Next, we tested whether circ_0008255 could affect ATG2A expression by sponging miR-192-5p. As expected, the levels of ATG2A mRNA and protein expression were significantly decreased after circ_0008255 was knocked down in the RPMI8226 and NCI-H929 cells (Fig. 6H and I). Importantly, co-transfection of synthetic miR-192-5p inhibitor could reverse the effect of sh-circ_0008255 on ATG2A mRNA and protein levels (Fig. 6H and I). When circ_0008255 was overexpressed, it induced the expression of ATG2A. However, co-transfection of synthetic miR-192-5p mimics impaired the impact of circ_0008255 overexpression on ATG2A mRNA (Fig. S7).

Previous work has demonstrated that Autophagy related 2 homolog A (ATG2A) is a critical protein for autophagosome formation and lipid droplets phagocytosis, but the precise mechanism underlying its role in autophagy is not clear [17]. For this reason, we analyzed the effect of ATG2A on the autophagy activity in MM cells. As expected, RPMI8226 and NCI-H929 cells with ATG2A knockdown showed lower LC3B-II and higher SQSTM1 levels than those of control cells (Fig. S8D), and autophagic flux analysis showed LC3B dots decreased in the MM cells transfected with ATG2A-shRNA, either with or without BAF treatment (Fig. S8E). Importantly, CCK-8 analysis showed that ATG2A knockdown significantly reduced the cell viability of MM cells (Fig. S8F), and colony formation assay and EdU assay indicated that ATG2A knockdown inhibited the proliferation ability of MM cells (Fig. S8G–I). Altogether, these data indicate that ATG2A is a direct target gene of miR-192-5p, which functions as an oncogene in MM, likely by mediating autophagy.

We then validated the role of ATG2A in MM cell autophagy in the context of its regulation by miR-192-5p and circ_0008255. As shown in Fig. S8J, similar to circ_0008255 knockdown, silencing of ATG2A with shRNA significantly reduced the LC3B-II:LC3B-I ratio and SQSTM1 degradation in both RPMI8226 and NCI-H929 cells. Simultaneously, ectopic expression of ATG2A rescued the autophagy-inhibiting effect of miR-192-5p, after BAF treatment (Fig. S8K). Furthermore, although circ_0008255 knockdown partially downregulated its downstream ATG2A, abundant ATG2A still persisted in cells. Additional silencing of ATG2A in circ_0008255-deficient cells exerted a true synergistic effect on autophagy inhibition, as evidenced by altered LC3B puncta, LC3B-II conversion and SQSTM1 accumulation. (Fig. 6J–K, Fig. S9). To confirmed ATG2A as a necessary downstream effector, a combination experiment with ATG2A knockdown and circ_0008255 overexpression was performed, As shown in Fig. S10, the enhanced autophagic activity induced by circ_0008255 overexpression was completely abrogated when ATG2A was simultaneously silenced. Therefore, the miR-192-5p/ATG2A axis mediates the autophagy-inducing activity of circ_0008255 in MM cells.

Finally, we employed a MM cell-derived xenograft mouse model to elucidate the therapeutic potential of targeting circ_0008255 and/or ATG2A. Xenografted mice were randomized to receive 4 different treatments when palpable tumors became detectable. As shown in Fig. 6L–O, tumor growth decreased in mice treated with circ_0008255-targeting shRNA (sh-circ_0008255) or ATG2A-targeting shRNA (sh-ATG2A), alone or in combination, in comparison with control mice. Notably, the combination of sh-circ_0008255 and sh-ATG2A exerted a more significant effect than either alone in suppressing tumor growth. Immunohistochemical staining of the harvested tumors revealed that circ_0008255 knockdown or ATG2A silencing significantly decreased autophagy marker LC3B-II and the proliferation biomarker Ki67. Moreover, the combination therapy of sh-circ_0008255 and sh-ATG2A further downregulated the expression of LC3B-II and Ki-67 (Fig. 6P). Western blot analysis yielded results consistent with IHC staining (Fig. 6Q). Additionally, we performed rescue experiments both in vitro and in vivo to verify whether miR-192-5p inhibition or ATG2A overexpression could reverse the anti-proliferative phenotype caused by circ_0008255 knockdown. In vitro CCK-8 results confirmed that stable circ_0008255 silencing markedly suppressed the proliferation of RPMI8226 and H929 cells. Importantly, either inhibition of miR-192-5p via miR-192-5p inhibitor or ectopic overexpression of ATG2A successfully rescued the proliferation arrest triggered by circ_0008255 depletion (Fig. S11A). Consistent with cellular data, our in vivo xenograft rescue assays further validated this regulatory axis: sh-circ_0008255 treatment dramatically retarded subcutaneous tumor growth and yielded the minimal tumor volume relative to the sh-ctrl group. Intratumoral administration of miR-192-5p antagomir or ATG2A-overexpressing adenovirus effectively abrogated the tumor-suppressive effect of circ_0008255 knockdown and restored tumor proliferation in vivo (Fig. S11B–E).

Overall, these results recapitulate the in vitro observations and indicate that disruption of the function of circ_0008255 and ATG2A is a promising therapeutic approach for the treatment of MM by autophagy inhibition.

### 2.7 Construction of a comprehensive autophagy blockade system inhibits multiple myeloma cell proliferation and autophagic

**Fig. 7:**
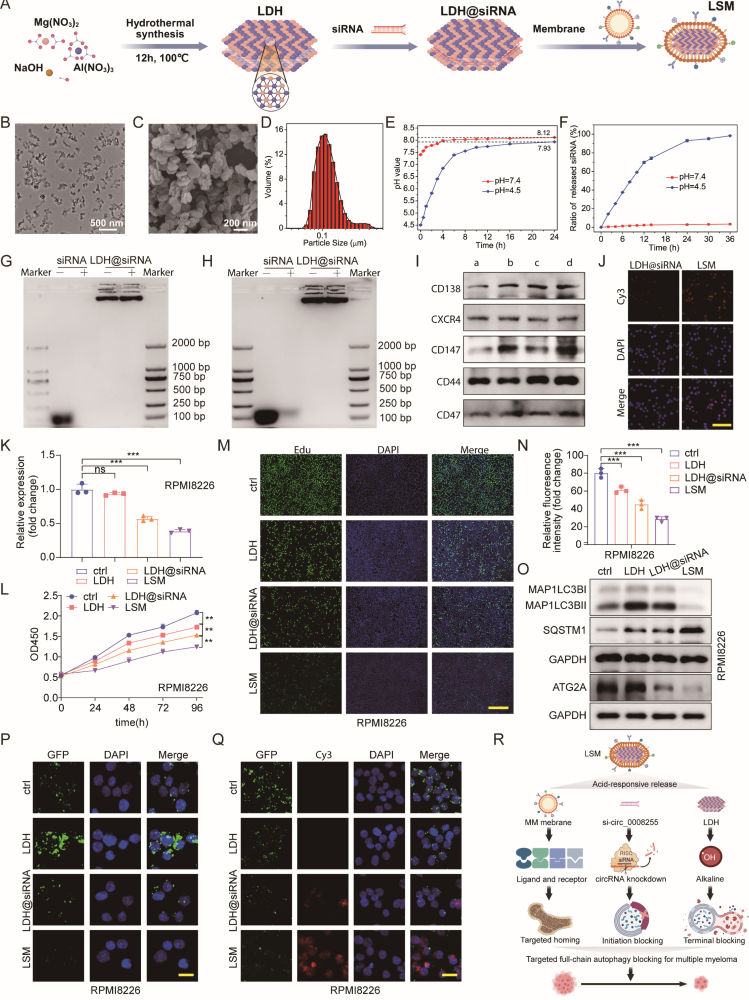
**Characteristics and antitumor effects of LSM in MM cells in vitro**. **A)** Process diagram for material synthesis. Panel **a** partially created with BioRender; **B)** TEM; **C)** SEM; **D)** DLS; **E)** The effect of LDH@siRNA degradation on the pH value of the solution; **F)** Release curves of siRNA under different pH conditions; **G)** Free siRNA (2 μg) and LSM was treated with 2 U/μg of RNase R at 37°C. Then agarose gel electrophoresis assay was performed to detect the RNA. **H)** Free siRNA (2 μg) and LSM was treated with human serum at 37°C. Then agarose gel electrophoresis assay was performed to detect the RNA**. I)** Western blot assay was performed to detect the CD138, CXCR4, CD147, CD44 and CD47. The samples were divided into 4 groups: a. RPMI8226 cell lysis group, b. membrane of RPMI8226, c. multiple myeloma vesicles, d. multiple myeloma cells treated with extrusion. **J,** Intracellular delivery of LDH@siRNA nanosheets and LSM nanosheets on RPMI8226 cells after 4 h incubation observed by CLSM (Scale bar = 100 μm). **K)** RT-qPCR assays were performed to detect the expression of circ_0008255 in RPMI8226 with different treatment. **L)** Cell proliferation changes in RPMI8226 measured by CCK-8 assay; **M,N)** Cell proliferation changes in RPMI8226 assessed by EdU staining (Scale bar = 500 μm). **O)** Western blot assay was performed to detect the expression of ATG2A, LC3B and SQSTM1. **P)** Confocal microscopy observation of cell autophagy in RPMI8226 stably expressing GFP-LC3B (Scale bar = 25 μm). **Q)** Lysosomal staining is performed using lysosomal probes in RPMI8226 with different treatment. (GFP: Lysosomal; Cy3: siRNA). **R)** Schematic diagram of the action process of LSM nanosheets. Panel **R** partially created with BioRender. All the data are presented as the mean ± standard deviation from 3 independent experiments, n.s. *P > *0.05, *P < 0.05, **P < 0.01, ***P < 0.001.

Given the critical role of circ_0008255 in promoting MM by inducing autophagy, we sought to develop an efficient siRNA delivery system to inhibit circ_0008255, thereby suppressing autophagy as a potential therapeutic strategy for MM. Layered Double Hydroxides (LDH) were selected as the delivery vector due to their favorable properties—high loading capacity, effective siRNA protection, and responsive release—that collectively enable efficient gene silencing. First, Mg/Al LDH nanosheets were prepared via hydrothermal method (Fig. 7A), followed by siRNA loading into LDH interlayers and surface coating with MM cell membranes to construct the biomimetic LDH@siRNA@MM (LSM) nanomaterial [18]. TEM, SEM, and DLS characterization confirmed LSM nanosheets exhibited a sheet-like morphology with an approximate size of 100 nm (Fig. S12A-C, Fig. 7B–D). High-resolution TEM revealed clear lattice fringes with a spacing of 1.542 Å (corresponding to the (223) crystal plane of LDH) (Fig. S12D), and selected area electron diffraction (SAED) demonstrated a polycrystalline structure (Fig. S12E). XRD data shows that siRNA loading does not alter LDH's crystal structure (Fig. S12F). Elemental mapping and XPS verified the presence of surface elements C, N, O, Mg, Al, and P (Fig. S12G–K), while FTIR spectra of LDH@siRNA displayed characteristic absorption peaks of LDH (1384 cm^−1^) and siRNA (1078 cm^−1^, 1238 cm^−1^) (Fig. S12L), confirming successful LSM fabrication. Meanwhile, we studied the mechanism underlying the stable coating of MM cell membranes on the surface of LDH nanosheets. As shown in Fig. S12M, the zeta potential of the LDH@siRNA nanosheets is +39.33 mV, indicating a high positive surface charge. It is well known that the MM cell membrane is overall negatively charged due to the presence of phospholipids (e.g., phosphatidylserine) and membrane proteins [19]. Therefore, strong electrostatic attraction between the positive and negative charges enables the LDH@siRNA nanosheets to tightly bind with the MM cell membrane, forming stable LSM nanocomposites. In addition, the rigid two-dimensional structure of the LDH nanosheets provides physical support for the membrane, further enhancing the stability of the composite. Subsequently, we evaluated the siRNA loading capacity of LDH by UV–vis absorption spectroscopy. The experimental results showed that the mass percentage of siRNA loaded in LSM nanosheets was 1.24% (Fig. S12N–P). Then, the pH-modulating capacity of LSM was evaluated using a pH meter. When added to acidic solution (pH = 4.5), LSM gradually increased the solution pH to 7.93 over time (Fig. 7E), siRNA release performance was assessed by labeling siRNA with Cy3 fluorescent dye. Release curves showed negligible siRNA release under neutral conditions (pH = 7.4), while significant release was observed under acidic conditions (pH = 4.5) (Fig. 7F and Fig. S12Q).

To assess the protective effect of LDH nanosheets on siRNA, we treated LDH@siRNA complexes and free siRNA with RNase and human serum. Agarose gel electrophoresis revealed that while free siRNA was completely degraded, the LDH-loaded siRNA remained intact (Fig. 7G). A consistent result was observed in human serum (Fig. 7H), indicating that LDH loading significantly enhances siRNA stability. We next evaluated the integrity of key membrane proteins (CD44, CD147, CXCR4, CD138, CD47) critical for myeloma cell homing (CD44, CD147, CXCR4), targeting (CD138), and immune evasion (CD47). Western blot analysis confirmed their stable presence in RPMI8226 cells, their membrane lysates, derived vesicles, and extruded myeloma cells, confirming that the membrane preparation process preserved protein integrity. Furthermore, these proteins were successfully retained on LSM nanosheets (Fig. 7I). To verify the cell membrane coating on nanosheets, we co-incubated the formulations with RPMI8226 cells. The LSM nanosheets group exhibited higher cellular fluorescence intensity than the LDH@siRNA group (Fig. 7J, Fig. S13A), demonstrating enhanced cellular association due to the membrane coating.

To clarify LSM's myeloma-specific targeting, we used high-marker RPMI8226, low-marker U266 myeloma cells and MHCC97H hepatoma control cells. Western blot showed CD44/CD147/CXCR4 expression ranked RPMI8226 > MHCC97H > U266 (Fig. S13B). Cy3 uptake assay demonstrated LSM internalization followed RPMI8226 > U266 ≫ MHCC97H (Fig. S13C–D). CCK-8 assays revealed strong proliferation inhibition in both myeloma lines but weak suppression in MHCC97H (Fig. S13E). Collectively, LSM relies on full myeloma membrane proteome for homotypic recognition instead of single markers, enabling effective targeting of low-marker myeloma cells and limited off-target effects on non-myeloma cells with CD44/CD147/CXCR4 expression.

To evaluate the impact of LSM on circ_0008255 expression and autophagy in multiple myeloma (MM) cells, we treated cells with LDH, LDH@siRNA, and LSM. RT-qPCR analysis revealed that LDH alone did not affect circ_0008255 levels, whereas LSM induced more potent silencing than LDH@siRNA (Fig. 7K, Fig. S13F). Functionally, LSM significantly suppressed MM cell proliferation, as confirmed by CCK-8 and EdU assays (Fig. 7L–N, Fig. S13G–I). WB analysis was employed to dissect the autophagic response. LDH treatment increased both LC3B-II and SQSTM1 levels, indicating blockade of autophagic flux. This is consistent with LDH-induced neutralization of the lysosomal acidic environment, which inactivates acid hydrolases and impairs autophagosome degradation. In contrast, LDH@siRNA downregulated LC3B-II but increased SQSTM1, suggesting inhibition of autophagosome initiation. LSM exhibited a similar but stronger regulatory pattern (Fig. 7O, Fig. S13J). Supporting these findings, immunofluorescence imaging showed that LDH increased GFP-LC3B puncta formation, while LSM reduced it (Fig. 7P and Fig. S13K–L). Furthermore, lysosomal staining revealed that LSM treatment led to a decrease in lysosomal number and a dispersed distribution (Fig. 7Q, Fig. S13M–N), confirming lysosomal dysfunction. To quantitatively verify LDH-mediated lysosomal alkalinization, we conducted ratiometric pH detection with LysoSensor Yellow/Blue DND-160 across distinct treatment groups. Quantitative fluorescence results demonstrated that LSM induced markedly more prominent lysosomal alkalinization relative to bare LDH nanosheets (Fig. S13O). Furthermore, to clarify whether the combined anti-myeloma effect is synergistic or merely additive, we set serial gradient concentrations for single LDH, single circ_0008255 siRNA, and LDH@siRNA nanocomplex at a fixed mass ratio (LDH:siRNA = 100:1.24). As shown in Fig. S14 and Table S4, the CI values at ED50, ED75 and ED90 were 0.32444, 0.43265 and 0.57762 respectively, all far below 1. According to classic Chou-Talalay criteria, CI < 1 firmly confirms prominent synergism rather than simple additive effect.

Collectively, these results demonstrate that LSM enables a full-chain blockade of autophagy, as shown in Fig. 7R: the released siRNA inhibits circ_0008255 to suppress autophagosome formation at the early stage, while the LDH nanosheet component alkalinizes lysosomes and disrupts autophagosome-lysosome fusion at the terminal stage. This dual mechanism, facilitated by the acidic microenvironment-responsive release of siRNA and the pH-modulating capability of LDH, provides an innovative strategy for autophagy inhibition in MM therapy.

### 2.8 LSM nanosheets mediate targeted bone marrow delivery and robust inhibition of tumor growth and bone destruction in vivo

**Fig. 8:**
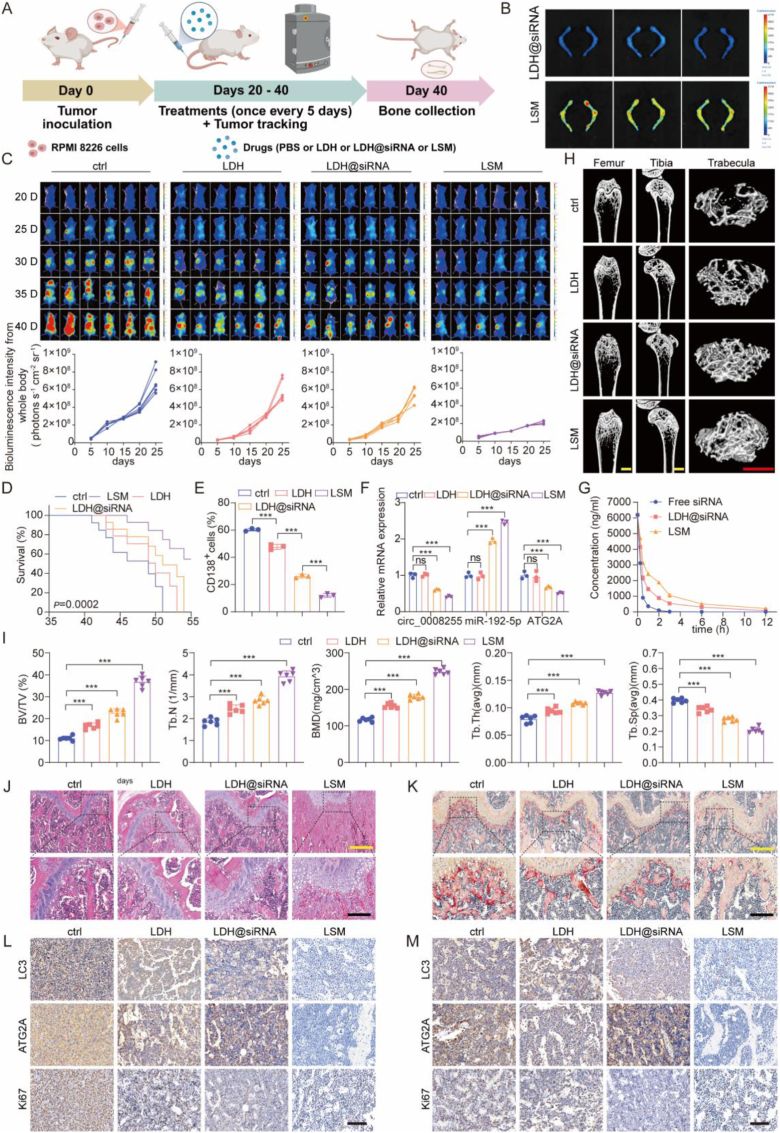
**In Vivo Efficacy of LSM: Suppression of Tumor Growth and Inhibition of Bone Lesions**. **A)** A schematic illustration of the experimental design; **B)** Ex vivo fluorescence imaging analysis of femoral tissues from NOG mice at 48 h after injection. **C)** Bioluminescence images of mice treated with saline (control, ctrl), LDH, LDH@siRNA, and LSM. **D)** Kaplan-Meier survival curves of tumor-bearing mice. **E)** Statistical chart of CD138^+^ cell proportions. **F)** RT-qPCR analysis for detecting expression changes of circ_0008255, miR-192-5p and ATG2A. **G)** Plasma siRNA concentration-time profiles of free siRNA, LDH@siRNA and LSM within 12 h. **H)** Micro-CT images (Yellow scale bar = 1 mm. Red scale bar = 1 mm). **I)** Quantitative analysis of BV/TV, Tb.N, BMD, Tb.Th, Tb.Sp (n = 6). **J)** HE staining of femur from NOG mice with different treatments (Yellow scale bar = 500 μm. Black scale bar = 100 μm). **K)** TRAP staining of fermur from NOG mice with different treatments (Yellow scale bar = 500 μm. Black scale bar = 100 μm). **L, M)** IHC staining of LC3B, ATG2A and Ki67 in femur(h) and tibia(i) (Scale bar = 100 μm). n.s. *P > *0.05, *P < 0.05, **P < 0.01, ***P < 0.001.

Building on our previous findings confirming that LSM effectively suppresses multiple myeloma cell proliferation and autophagy through full-chain autophagy blockade, we further evaluated its in vivo targeting capability and antitumor efficacy. An orthotopic multiple myeloma model was established by tail vein injection of RPMI8226-luciferase cells Fig. 8A. Model mice were treated with LDH@siRNA or LSM formulations for 48 h, after which they were euthanized, and major organs and femoral tissues were collected for ex vivo imaging. Results revealed that the LSM group displayed the highest fluorescence intensity in bone marrow, whereas no significant intergroup differences were observed in other tissues (Fig. 8B, Fig. S15A–B), indicating that the cell membrane coating is essential for conferring bone marrow homing and tumor-targeting properties to LSM nanosheets. Furthermore, bioluminescence tracking over 20 days under different treatments showed that the LSM-treated group developed significantly fewer metastatic lesions compared with the control group (Fig. 8C). Importantly, compared with the control group, LSM significantly improved the overall survival of multiple myeloma model mice (Fig. 8D, Fig. S15C). Furthermore, we isolated CD138^+^ MM cells from mouse bone marrow at day 40 of the in vivo experiment and performed RT-qPCR to quantify the transcript levels of circ_0008255, miR-192-5p and ATG2A. The results demonstrated that the LSM treatment group exhibited the most prominent downregulation of circ_0008255 and ATG2A, accompanied by the strongest upregulation of miR-192-5p among all treatment groups (Fig. 8E–F, Fig. S15D). This finding recapitulates our in vitro regulatory axis data in primary tumor cells within the bone marrow niche. Then the pharmacokinetics of the free siRNA, LDH@siRNA and LSM were evaluated by measuring the drug concentration in the plasma (Fig. 8G, Fig. S15E), The results directly validate the superior in vivo delivery performance of biomimetic LSM over free siRNA and uncoated LDH@siRNA. These results collectively demonstrate that LSM effectively inhibits myeloma metastasis and improves overall mouse survival condition.

Osteolysis is a hallmark clinical manifestation of multiple myeloma. To assess bone destruction, we performed micro-computer tomography (micro-CT) on murine femurs. The results revealed severe bony destruction and trabecular bone loss in the control group, whereas the LSM-treated group exhibited a significant reduction in osteolytic damage (Fig. 8H). Subsequent quantitative micro-CT analysis further demonstrated that LSM treatment led to marked improvements in key bone structural parameters, including increases in bone volume fraction (BV/TV), trabecular number (Tb.N), bone mineral density (BMD), trabecular thickness (Tb.Th), and bone surface area, alongside a decrease in trabecular separation (Tb.Sp) (Fig. 8I). Consistently, HE staining results showed that compared with the control group, the mice in the LSM-treated group had significantly increased cortical bone thickness and remarkably reduced bone destruction (Fig. 8J, Fig. S15F). TRAP staining further revealed a significant decrease in osteoclast numbers following LSM treatment, confirming its efficacy in inhibiting bone destruction in the MM mouse model (Fig. 8K, Fig. S15G). Finally, IHC analysis showed that LSM treatment significantly downregulated the expression of LC3B, SQSTM1, and Ki67 in mouse tissues, indicating potent in vivo inhibition of autophagy and attenuation of tumor cell proliferation (Fig. 8L and M).

Having established from histological staining that no major organs exhibited obvious pathological changes across treatment group (Fig. S16A), we further evaluated the systemic safety of the nanoformulations. Analysis of peripheral blood collected seven days post-injection showed that key hematopoietic parameters—including lymphocytes, hematocrit (Hct), neutrophils, and platelet counts—remained largely unaffected in all groups (Fig. S16B–E). Furthermore, blood biochemical analyses revealed no significant signs of toxicity (Fig. S16F–J). To evaluate autophagic levels in non-skeletal peripheral organs at the experimental endpoint, heart, liver, spleen, lung and kidney tissues were harvested from all four groups for LC3B immunohistochemistry. No statistically significant differences in LC3B expression were observed among ctrl, LDH, LDH@siRNA and LSM groups across all examined organs, verifying that our treatment regimens do not perturb physiological autophagy in normal peripheral tissues (Fig. S17). Together, these data confirm that the multiple myeloma-mimetic cell membrane-coated nanosheets (LSM) not only successfully home to the bone marrow but also do not impair normal hematopoietic function. Overall, LSM nanosheets effectively inhibit MM proliferation and metastasis in vivo while exhibiting a favorable safety profile, underscoring their promising therapeutic potential for multiple myeloma.

### 2.9 Discussion

Circular RNAs (circRNAs) have emerged as promising targets for diagnosing and treating human diseases, owing to their stable structure and cell-type-specific expression patterns [20]. In this study, we identified and characterized a novel MM-associated circRNA, circ_0008255, which originates from the 21st and 22nd exons of the parent gene ABCC1. Clinically, circ_0008255 is highly expressed in MM patients and closely associated with poor disease prognosis (Fig. 2). Intriguingly, we demonstrate that knockdown of circ_0008255 inhibited MM cellular proliferation and tumor growth, and suppressed autophagy of MM cells (Fig. 3, Fig. 4). Mechanistically, we showed for the first time that circ_0008255 upregulated the expression of the autophagy-related gene ATG2A by sponging miR-192-5p, thus promoting autophagy and tumor growth (Fig. 5, Fig. 6). Next, in a MM xenograft mouse model, depletion of endogenous circ_0008255 and ATG2A alleviated progression by autophagy inhibition (Fig. 6). Finally, building on these mechanistic insights, we achieve full-chain autophagy-blocking therapy for multiple myeloma by encapsulating LDH-loaded siRNA in MM cell membranes (Fig. 7, Fig. 8).

Accumulating evidence has indicated that dysregulated circRNAs are implicated in various human cancers [21]. However, to date, few circRNAs have been reported to regulate autophagy in the pathobiology of MM [[22], [23], [24]]. To the best of our knowledge, the present study is the first to uncover a novel autophagy-associated circRNA in MM, circ_0008255, which is elevated in MM patients, predicts poor prognosis and modulates autophagy during MM progression.

Autophagy is a highly conserved catabolic process, which is initiated and tightly regulated by a suite of autophagy-related genes (ATGs) [25]. As a member of the ATG family, ATG2A is recognized as a critical regulator during the early stages of autophagosome formation, thereby facilitating autophagosome membrane construction and participating in the expansion of the autophagosomal membrane in various cancers [26]. However, the role of ATG2A in malignancies, including MM, remains unclear. In the present study, we provided the first evidence that ATG2A was upregulated in human MM, and its silencing significantly inhibits the malignant phenotypes of MM cells. These findings support an oncogenic role of ATG2A in MM and suggest that ATG2A represents another potentially novel therapeutic target for MM. Accumulated evidence demonstrates that inhibiting merely one step of autophagy cannot thoroughly suppress pro-survival autophagic flux in tumor cells. A recent authoritative review systematically outlined the drawbacks of single-stage autophagy inhibition: tumor cells activate NRF2 antioxidant signaling, microautophagy and compensatory upregulation of ATG family proteins to counteract incomplete autophagy arrest, leading to weak and transient anti-tumor effects for single autophagy inhibitors such as 3-MA and hydroxychloroquine [27]. To overcome this limitation, we constructed LSM biomimetic nanoparticles to realize full-chain autophagy blockade by synchronously disrupting early autophagosome biogenesis and late lysosomal degradation. The dual inhibitory arms jointly eliminate the compensatory survival pathways activated upon single-node intervention, achieving complete interruption of autophagic flux and synergistic anti-myeloma activity, which distinguishes our strategy from conventional single-target autophagy interventions reported previously.

Our findings address a significant unmet clinical need. Recent evidence suggests that circRNAs can serve as potential biomarkers or therapeutic targets [6]. We showed that upregulated circ_0008255 is closely associated with disease progression and poor prognosis, indicating that circ_0008255 as a potential diagnostic and prognostic biomarker for MM. Till now, MM remains an incurable disease due to clonal heterogeneity [28]. In this study, we provided in vitro and in vivo pre-clinical experiment data to demonstrate that targeting the circ_0008255/miR-192-5p/ATG2A axis holds the potential for the treatment of MM via inhibiting MM autophagy.

In recent years, nanoencapsulation technology has exhibited unique advantages in two key aspects: protecting encapsulated payloads from degradation in the biological microenvironment and enabling precise targeted delivery [[29], [30], [31]]. Layered Double Hydroxide (LDH) nanomaterials exhibit multi-dimensional advantages in the field of drug delivery due to their unique physicochemical and biological properties. Their high loading capacity, pH responsiveness, biocompatibility, and immunomodulatory potential have been verified in various models of diseases such as cancer and cardiovascular diseases [32]. In previous studies conducted by our group, AZ-23 was loaded onto LDH to block tumor-nerve crosstalk, successfully inhibiting tumor growth and alleviating bone pain in bone metastasis models [33]. In the present study, we employed LDH for siRNA delivery. Specifically, LDH enhanced siRNA stability while inhibiting autolysosome formation. LDH possesses weak alkaline buffering capacity. After the LDH/siRNA complexes are endocytosed into acidic endosomes/lysosomes (pH 4.5–5.5), the LDH nanosheets gradually dissolve and release OH^−^, which neutralize H^+^ in the lumen, leading to lysosomal alkalinization. To restore the acidic environment, V-ATPase continuously pumps H^+^ into the lumen, but this process is constantly counteracted by the buffering effect of LDH, resulting in futile proton cycling (the proton sponge effect). As Cl^−^ influx occurs to maintain electroneutrality, the osmotic pressure inside the vesicle increases, causing water influx. Eventually, the lysosome swells and ruptures, thereby blocking autophagosome-lysosome fusion, inhibiting autophagolysosome formation, and simultaneously releasing siRNA into the cytoplasm, where the released siRNA further suppresses autophagosomal membrane fusion. By encapsulating MM cell membranes to augment targeting specificity, this system ultimately enabled a full-chain autophagy-blocking therapy for MM. In translational perspective, LSM relies on myeloma homotypic recognition to selectively accumulate in CD138^+^ malignant plasma cells, restricting autophagy inhibition to tumor cells rather than triggering global systemic autophagy suppression. Moreover, the human-specific feature of circ_0008255 minimizes the risk of disrupting autophagy homeostasis in healthy human tissues, supporting the favorable safety profile of this biomimetic nanotherapy for clinical application.

The homing of MM cells to the bone marrow (BM) niche is a defining hallmark of the disease, playing a central role in tumor seeding, progression, and acquired drug resistance [34]. Leveraging this natural tropism, we fabricated a biomimetic LSM nanoplatform coated with myeloma cell membranes. The outer myeloma membrane shell facilitates bone marrow tropism and elevates specific cellular uptake and intracellular delivery in myeloma cells. LSM exerts synergistic autophagy suppression via two coordinated pathways: encapsulated siRNA inhibits early autophagosome formation through the circ_0008255/ATG2A axis, while internal LDH triggers lysosomal alkalinization to block late-stage autophagosome–lysosome fusion. CalcuSyn combination index analysis quantitatively validates this synergistic anti-autophagy effect. Attributed to homologous membrane-mediated bone marrow targeting, LSM achieves stronger anti-myeloma efficacy than uncoated LDH@siRNA.

While this study provided valuable insights into the role of circ_0008255 in MM pathogenesis and offers a novel therapeutic strategy, several limitations and areas for further improvement should be acknowledged. First, to further enhance the targeting specificity of LSM for MM cells, CD38 antibodies (a clinically validated MM-targeting agent [35]) could be conjugated to the surface of the MM cell membrane coating, potentially improving BM niche accumulation and reducing off-target effects. Additionally, high autophagic activity has been shown to mediate MM cell resistance to bortezomib, a frontline therapeutic for MM [36]. Co-loading bortezomib into the LSM nano-system could therefore enable synergistic therapy, combining autophagy inhibition with direct cytotoxicity to overcome drug resistance. Addressing these limitations will not only strengthen the translational potential of our findings but also accelerate the development of circ_0008255-targeted and autophagy-blocking therapies for MM.

## 3 Conclusion

This study discovered circ_0008255 as a novel autophagy-related circRNA, characterized its functional role and clinical significance in MM pathogenesis, and elucidated it exerted its effects through the miR-192-5p/ATG2A axis. Furthermore, we developed a novel therapeutic system targeting circ_0008255, enabling full-chain autophagy-blocking therapy for MM. Collectively, our findings may provide insights into the identification of novel biomarkers and therapeutic targets for MM.

## Data availability statement

All data generated or analyzed during this study are included in the article and its supplementary information files or are available from the corresponding author upon reasonable request.

## Ethics approval and consent to participate

All participants in this study provided written informed consent and the study was approved by the Institutional Research Ethics Committee at Changzheng Hospital (Shanghai, China) (approval number: No. 2021SL034, 2023SLYS7).

## Funding

This study was supported by 10.13039/501100001809National Natural Science Foundation of China (82402683, 82402695, 82372313, 82272390, 82102482, 82072371, 82573332, U25A20245), Program of Shanghai Academic/Technology Research Leader (Grant No.23XD1404900), Shanghai Healthcare Commission Young Talent Program (2022YQ056), and 10.13039/501100003399Shanghai Science and Technology Committee under Grant (21ZR1478200).

## CRediT authorship contribution statement

**Zhenhua Wang:** Conceptualization, Data curation, Formal analysis, Funding acquisition, Investigation, Methodology, Project administration, Writing – original draft. **Hefei Ren:** Data curation, Funding acquisition, Validation, Writing – original draft. **Kun Wang:** Formal analysis, Investigation, Methodology. **Xiaomin Zhang:** Data curation, Investigation, Resources. **Chang Liu:** Funding acquisition, Visualization. **Hongkun Wu:** Funding acquisition, Resources. **Jieyun Shi:** Formal analysis, Methodology. **Jiafeng Zhang:** Resources. **Chaochao Wang:** Methodology, Project administration. **Liuzhi Wu:** Data curation. **Juan Du:** Data curation, Investigation, Resources, Validation. **Fenyong Sun:** Conceptualization, Project administration, Supervision. **Yelin Wu:** Formal analysis, Investigation, Supervision, Writing – review & editing. **Lin Zhou:** Conceptualization, Funding acquisition, Supervision, Writing – original draft, Writing – review & editing.

## Declaration of competing interest

The authors declare that they have no competing interests.
