# Supplementary material for: LDH-mediated autophagic full-chain blockade for Multiple Myeloma treatment by targeting circ_0008255/miR-192-5p/ATG2A axis
Source: Bioact Mater. 2026 Aug 4;67:127–42. doi: 10.1016/j.bioactmat.2026.07.054 (PMC13471063; doi:10.1016/j.bioactmat.2026.07.054)
Supplement: Supplementary file 1 [file mmc1.docx]

**1. Methods and materials**

**1.1. Clinical patient samples**

Following informed consent and the approval of the Institutional Research Ethics Committee at Changzheng Hospital (Shanghai, China) (approval number: No. 2021SL034, 2023SLYS7), we collected a total of 121 patients diagnosed with multiple myeloma (MM) from May 2019 to December 2019. The diagnosis of MM was based on the guidelines outlined in the "Chinese Guidelines for the Diagnosis and Treatment of Multiple Myeloma (2017 Revised Edition)" ^1^. Among the 61 newly diagnosed patients, there were 29 males and 32 females. Out of these, 28 cases were classified as IgG subtype, 12 cases as IgA subtype, 4 cases as IgD subtype, 15 cases as light chain subtype, and 2 cases as non-secretory subtype. The patients were also classified according to the International Staging System (ISS), with 10 cases in ISS stage I, 17 cases in ISS stage II, and 34 cases in ISS stage III. More detailed clinical characteristics of the 61 newly diagnosed patients can be found in Table S1 and Table S2. These patients were followed up for a period of 4 years. Our study included 80 healthy controls recruited from the blood donation center of Shanghai Changzheng Hospital. Plasma cells from MM patients and normal controls were purified from bone marrow aspirates by antibody mediated positive selection using anti-SDC1/CD138 magnetic activated cell separation microbeads (Miltenyi Biotec, 130-051-301). Plasma cell purity was tested to be 98% (> 90%). Peripheral blood plasma and bone marrow samples were collected from both the patients and healthy controls, then were divided into aliquots of 500 μl and stored at -80°C.

**1.2. Cell lines and cell culture**

As in our previous study ^2^, human MM cell lines RPMI8226, U266, NCI-H929 were purchased from American Type Culture Collection (ATCC), while LP-1 and KM3 were kindly provided by Dr. Jian Hou (Naval Medical University, China). All MM cell lines, as well as plasma cells from healthy individuals, were cultured in RPMI1640 containing 10% serum and 1% antibiotics. The cells were maintained at 37 °C in a humidified atmosphere with 5% carbon dioxide.

**1.3. RNA sequencing**

CircRNA sequencing: This part of the sequencing was assisted by Shanghai BioHelper. Briefly, the following steps were conducted: Total RNA was extracted from the bone marrow samples of 3 patients and 3 healthy individuals using TRIzol reagent (Takara, Japan). The total RNA underwent rRNA depletion, RNase R digestion, fragmentation, first-strand cDNA synthesis, second-strand cDNA synthesis, end repair, 3’-end adenylation, adapter ligation, and enrichment to construct the sequencing sample library. Cluster generation and hybridization of the first sequencing primers were completed on the cBot system associated with Illumina sequencers. Sequencing reagents were prepared according to the Illumina User Guide, and the flow cell containing clusters was loaded onto the sequencer. Paired-end sequencing was performed, and the sequencing process was controlled by Illumina's data collection software with real-time data analysis.

Transcriptome sequencing: This portion of the sequencing was conducted with assistance from Hangzhou Lianchuan Biotech. The procedure involved extracting total RNA from processed MM cells using TRIzol reagent (Takara, Japan). For RNA-seq, the TruSeq Stranded mRNA LT Sample Prep Kit (Illumina, USA) was utilized for the preparation of strand-specific RNA-seq libraries. Quality control was performed using the Bioanalyzer 2100 (Agilent, USA), followed by sequencing on the NovaSeq 6000 platform (Illumina, USA). The raw reads of all samples were quality controlled using Trimmomatic software. The reference genome (HISAT2) software was used for spliced transcripts and sequence alignment of clean reads for each sample. Differential expression analysis was carried out using DESeq (2012) R package. Hierarchical clustering analysis of differentially expressed genes (DEGs) was conducted to display the expression patterns of genes in different groups and samples. Gene Ontology (GO) enrichment and Kyoto Encyclopedia of Genes and Genomes (KEGG) pathway enrichment analyses of DEGs were performed using R packages based on hypergeometric distribution.

**1.4. RNA extraction and RT-qPCR analysis**

According to the instructions, cell total RNA was extracted using TRIzol reagent (Takara), reverse transcribed into cDNA using the Ecrist AG (AG, 11706, China) reverse transcription kit, and the expression of circRNA and ATG2A was detected using the Ecrist AG (AG11701) RT-qPCR assay kit, with glyceraldehyde-3-phosphate dehydrogenase (GAPDH) serving as an internal reference gene. For the detection of miR-192-5p, we employed the "miRNA First-Strand cDNA Synthesis (Tailored Method)" kit (Sangon Biotech, B532451-0010) to generate miRNA templates, followed using the Ecrist AG (AG, 11701, China) RT-qPCR assay kit to detect the expression of miR-192-5p, with U6 as the internal reference gene. The primer sequences are shown in Table S5.

The full-length sequence of circ_0008255 as follows:

ACAGCTCAGCAGCTCCTCCTCCTATAGTGGGGACATCAGCAGGCACCACAACAGCACCGCAGAACTGCAGAAAGCTGAGGCCAAGAAGGAGGAGACCTGGAAGCTGATGGAGGCTGACAAGGCGCAGACAGGGCAGGTCAAGCTTTCCGTGTACTGGGACTACATGAAGGCCATCGGACTCTTCATCTCCTTCCTCAGCATCTTCCTTTTCATGTGTAACCATGTGTCCGCGCTGGCTTCCAACTATTGGCTCAGCCTCTGGACTGATGACCCCATCGTCAACGGGACTCAGGAGCACACGAAAGTCCGGCTGAGCGTCTATGGAGCCCTGGGCATTTCACAAG (344bps)

**1.5. Nucleus-cytoplasm fractionation, RNase R and Actinomycin D treatment**

RNA from nucleus and cytoplasm was separated using the PARIS™ Kit (Life Technologies, USA) following the manufacturer's instructions. Total RNA (2 μg) was treated with 2 U/μg of RNase R (Epicentre Biotechnologies, USA) at 37°C for 10, 20, 30, and 40 minutes, respectively. The RNA was then reverse transcribed into cDNA for subsequent experiments. To inhibit new RNA synthesis, cells were treated with 100 ng/ml Actinomycin D (CST, USA) for 24 hours, and the expression of circRNA and its parental gene was detected.

**1.6. FISH experiment**

MM cells were fixed with 4% paraformaldehyde (PFA) for 10 minutes, followed by overnight incubation in phosphate-buffered saline (PBS) at 4°C, and subsequent processing to detect the expression of circ_0008255 or miR-192-5p. The cells were then permeabilized with PBS containing 0.5% Triton X-100 for 15 minutes. After dehydration in 70%, 95%, and 100% ethanol for 5 minutes each, hybridization buffer containing FITC-labeled circ_0008255 probe (RiboBio, Guangzhou, China) and Cy3-labeled miR-192-5p probe (RiboBio) was heated at 88°C for 5 minutes, dropped onto the fixed cells, and then incubated overnight at 37°C in a dark humid chamber for hybridization. The next day, the cells were washed three times. Probe signals were detected following the instructions of the FISH kit (RiboBio). After washing three times with PBS, the cells were co-incubated with 4ʹ,6-diamidino-2-phenylindole (DAPI) (Santa Cruz Biotechnology, USA) at room temperature for 20 minutes to visualize the cell nuclei. Finally, the cells were observed using confocal microscopy. The probe sequence for h-circ_0008255 is 5’-GCTGCTGAGCT GTCTTGTGAAATGCCCA-3’, and the probe sequence for miR-192-5p is 5′-GGCTGTCAATTCATAGGTCAG-3′.

**1.7. Construction of stable MM cell lines**

The shRNA sequence targeting the circularization site of circ_0008255 was synthesized by OBiO (Shanghai, China). The synthesized sequence was inserted into the pCLenti-U6-shRNA-CMV-Puro-WPRE vector, and the recombinant plasmid was packaged into lentivirus and introduced into RPMI8226 and H929 cells. Positive clones were selected using 1 μg/ml puromycin, and the selected MM cells were used to establish stable circ_0008255 knockdown cell lines. The construction of stable ATG2A knockdown cell lines was performed in a consistent manner with the construction of stable circ_0008255 knockdown cell lines. The shRNA sequences are shown in Table S6.

**1.8. Cell transfection**

Human circ_0008255 sequence was inserted into the pLCDH-ciR overexpression vector to construct the circ_0008255 overexpression plasmid. Human ATG2A cDNA was inserted into the pcDNA3.1 vector to construct the ATG2A overexpression plasmid. The has-miR-192-5p miRNA mimics (miR10000222–1-5), inhibitor (miR20000222-1–5), and negative controls ((miR1N0000001-1-1) for mimic and (miR2N0000001-1–5) for inhibitor) were purchased from Ribobio. Transfection of synthetic miR-192-5p mimics, inhibitors, and corresponding controls was performed using Ribobio's transfection reagent (C10511). LipofectamineTM 3000 transfection reagent (Invitrogen, 13778-150) was used for the transfection of circ_0008255 overexpression plasmid and ATG2A overexpression plasmid.

**1.9. Cell proliferation assay**

Cell viability was assessed using the CCK-8 (Dojindo, CK04, Japan). shRNA controls and sh-circ_0008255 were separately transfected into MM cells, and the transfected MM cells were seeded in a 96-well plate and cultured at 37°C for 24, 48, 72, and 96 hours. After the necessary treatments, 10 μl of CCK-8 reagent was added to each well, followed by a 1.5-hour incubation at 37°C, and the absorbance value at OD 450 was measured using a microplate reader.

**1.10. EdU assay**

The EdU Assay Kit (Beyotime, C0071S, China) was used to detect DNA synthesis and cell proliferation. Firstly, 10,000 treated MM cells were seeded in a 96-well plate. The following day, the EdU solution (25 μM) was added to the wells and incubated for 24 hours. The cells were then fixed with 4% formaldehyde and permeabilized with 0.5% Triton X-100. Subsequently, the Apollo reaction solution was added to stain the EdU for approximately 30 minutes, while the cell nuclei were stained with Hoechst (33342). Finally, DNA synthesis and cell proliferation were observed using a Nikon microscope, with the signal reflected by red and blue colors respectively.

**1.11. Soft agar colony formation assay:**

Firstly, 1.2% and 0.7% Agarose were prepared in advance. After autoclaving, they were stored in a refrigerator at 4°C. On the day of plating, they were heated and melted and then placed in an incubator at 37 °C. 1.2% Agarose and RPMI1640 medium containing serum were mixed at a ratio of 1:1. Then, 2 ml of the mixture was taken for each well of a 6-well plate and left to cool and solidify at room temperature. Next, cells in the logarithmic growth phase were harvested and prepared into a single-cell suspension. After counting, the cell number was adjusted to 5×10⁴/ml. 0.7% Agarose and 20% RPMI1640 medium were mixed at a ratio of 1:1. 2 ml of the mixture was taken into a tube, and 10 - 20 μl of the cell suspension was added into the tube. After thorough mixing, the mixture was added to the 6-well plate with the bottom layer of agar already paved. After the upper layer of agar solidified, the plate was placed in an incubator at 37 °C for 2 weeks. During this period, 200 μl of medium was added to each well of the plate every three days. After 2 weeks, the medium was discarded. The cells were washed twice with PBS. Then, 4% PFA was added to fix the cells for 10 minutes. After that, the cells were washed twice with PBS again. Subsequently, crystal violet was added to stain the cells for 10 minutes. Finally, the cells were washed twice with PBS and photographed under a microscope.

**1.12. Western blot experiment**

The treated cells were collected in RIPA buffer (Beyotime, P1081) containing protease inhibitor and phosphatase inhibitor. Protein concentration was measured using the BCA assay kit. The extracted proteins were loaded onto a 12% SDS-PAGE gel at a quantity of 30 μg per well for electrophoresis. After electrophoresis, the proteins were transferred to a PVDF membrane. The membrane was then incubated with 5% non-fat milk at room temperature for 1 hour, followed by incubation with the corresponding primary antibodies overnight. Subsequently, the membrane was incubated with secondary antibodies, and imaging was performed using an imaging system. The primary antibodies used were SQSTM1 (CST, 5114), LC3B (CST, 3868), ATG2A (CST, 13509), CD138 (Protein tech, 10593), CXCR4 (Protein tech, 60042), CD147 (Protein tech, 11989), CD44 (Protein tech, 15675), CD47 (Protein tech, 20305).

**1.13. Luciferase reporter assay**

The wild-type and miR-192-5p binding site mutant of circ_0008255 were amplified using PCR. The amplified PCR products were then inserted into the pMIR-Report luciferase vector. The sequences were verified by next-generation sequencing. Recombinant plasmids, miRNA mimics, and β-gal plasmids were transfected into 293T cells. After 48 hours, the cells were lysed, and luciferase activity was measured using a dual-luciferase reporter gene assay kit (Promega, E1910) according to the manufacturer’s instructions. The wild-type and miR-192-5p binding site mutant of ATG2A 3'UTR were amplified using PCR. The amplified PCR products were then inserted into the pMIR-Report luciferase vector, following the same steps as described above.

**1.14. In vivo experiment**

A group of female BALB/c nude mice (5-6 weeks old) were purchased from the Shanghai Experimental Animal Center of the Chinese Academy of Sciences and housed in the specific-pathogen-free (SPF) animal facility at the Naval Medical University. All experimental procedures involving animals received ethical approval from the Animal Care and Use Committee of the Naval Medical University (approval number:2021SLYS04). To establish a xenograft model of human MM, 5×10^5^ stable circ_0008255 or ATG2A knockdown cells and 5×10^5^ corresponding control cells were separately mixed with 100 μl of matrix gel (Corning, 354248, USA), and injected subcutaneously into the mice. Tumor size was monitored and measured every 2 days starting from day 5 post-injection, and calculated using the formula: (length × width2) / 2. Upon reaching the twenty-first day post-inoculation, the mice were euthanized under anesthesia, following which the tumors were excised, fixed in 10% paraformaldehyde for preservation and subsequent analysis.

To establish the orthotopic multiple myeloma model, 5-6-week-old NOG mice were used (purchased from Hangzhou Ziyuan Laboratory Animal Technology Co., Ltd.). A total of 5×10⁵ RPMI8226 cells stably expressing luciferase were injected into the mice via the tail vein. 20 days after cell injection, the luciferase substrate was intraperitoneally injected into the mice, and thereafter, the tumor growth in the mice was monitored every 5 days using an in vivo imaging system. Concurrently, different intervention drugs were injected into the mice via the tail vein every 5 days, with the groups as follows: PBS control group, LDH group, LDH@siRNA group, and LSM group. On the 40th day of the experiment, the mice were euthanized, and relevant tissue samples were collected for microCT detection and immunostaining assays, respectively.

In vivo pharmacokinetics investigation: The pharmacokinetic detection procedure was performed in accordance with a previously published study in *Nature Communications* (2024)^3^. Peripheral blood was harvested via tail vein at preset time points (0, 0.25, 0.5, 1, 2, 3, 6, 12 h post-administration), and plasma was separated by centrifugation (15,000 × g, 10 min, 4 °C) in EDTA anticoagulant tubes and stored at −80 °C until use.Total RNA was extracted from 30 μL plasma using miRNeasy Serum/Plasma Kit (Qiagen). Stem-loop RT-qPCR was applied for absolute quantification of residual siRNA in plasma. A serial dilution gradient of pure target siRNA was mixed with blank mouse plasma to construct a matrix-matched standard curve, converting cycle threshold (Ct) values to absolute plasma siRNA concentrations. Custom stem-loop reverse transcription primers and amplification primers specific to circ_0008255 siRNA were used for RT-qPCR detection. Non-compartmental pharmacokinetic analysis was performed to calculate core pharmacokinetic parameters, including area under the concentration-time curve (AUC), plasma half-life (t₁/₂), plasma clearance (CL) and peak plasma concentration (Cmax). Each group contained n = 3 independent myeloma-bearing mice as biological replicates. RT-qPCR primer sequences for circ_0008255 siRNA are shown in Table S5

**1.15. Immunohistochemistry**

Immunohistochemistry was performed using the procedures specified in the immunohistochemistry kit (Abmart, A10026M, USA). Tumors derived from the subcutaneous tissue of nude mice were fixed overnight using 4% paraformaldehyde, followed by dehydration and embedding in paraffin. Thin sections were prepared and subjected to standard deparaffinization procedures until reaching water. An endogenous peroxidase blocking reagent was employed to deactivate endogenous enzymes and allowed to react for 10 minutes at room temperature. Subsequent washing steps with distilled water were performed for 2 minutes each, repeated three times. Antigen retrieval was carried out on the tissue sections. An endogenous peroxidase blocking agent was added to inactivate endogenous enzymes and incubated for 10 minutes at room temperature. The appropriately diluted primary antibody (Ki67 (CST, 9129), LC3B (CST, 12741), ATG2A (Protein tech, 23226-1)) was added and incubated overnight at 4 °C. The sections were washed with 0.01M PBS for 2 minutes, repeated three times. Polymer HRP-conjugated anti-rabbit IgG was applied to the sections and incubated at 37 °C for 30 minutes. The sections were then washed with PBS for 2 minutes, repeated three times. 3,3'-diaminobenzidine (DAB) staining was performed by taking 1ml distilled water and adding 50 μl each of the A, B, and C reagents from the kit. After thorough mixing, the mixture was applied to the sections for a 20-minute staining period, followed by rinsing with distilled water. Light counterstaining with hematoxylin was performed, followed by dehydration, clearing, mounting, and observation.

**1.16. Autophagy analysis**

Western blot analysis was performed to assess the expression levels of LC3B-I, LC3B-II, SQSTM1, and GFP-LC3B, revealing alterations in autophagic activity. Experiments were conducted under conditions with or without the addition of autophagy inhibitors such as 3-MA, BAF, and CQ). Stable cell lines expressing GFP-LC3B were established in RPMI8226, NCI-H929, and U266 cells using the Stub-RFP-Sens-GFP-LC3B lentiviral vector (GENECHEM, GVAP01689345, China). Subsequently, single miRNAs, siRNAs, or pcDNA were transfected into RPMI8226, NCI-H929, and U266 GFP-LC3B stable cells with or without lysosome inhibitor BAF (20 nM). The cells were fixed in 4% formaldehyde (Sangon Biotech, E672002) and permeabilized with 0.5% Triton X-100 (Thermo Fisher Scientific, 85,111, USA). Subsequent staining was carried out at room temperature in the dark DAPI. Spot formation of GFP-LC3B was visualized through image acquisition using inverted confocal fluorescence microscopy (Carl Zeiss, Germany).

**1.17. RNA immunoprecipitation (RIP) assay**

The RIP experiment was conducted following the instructions of the Magna RIP RNA Binding Protein Immunoprecipitation Kit (Millipore, USA). AGO2 and IgG were purchased from Abcam (ab5072, rabbit polyclonal antibody, USA). Total RNA was extracted and expression levels of circ_0008255 and miR-192-5p were detected using RT-qPCR method.

**1.18. RNA pull-down assay**

Biotin-labeled probes targeting circ_0008255 and scrambled negative-control oligo probes were synthesized by GenePharma (Shanghai, China). The biotinylated circ_0008255 probe was pre-incubated with C-1 streptavidin magnetic beads (Life Technologies, Waltham, MA, USA) at 25 °C for 2 h to immobilize probes onto bead surfaces. After cell collection and lysis, whole-cell lysates were incubated overnight at 4 °C with bead-conjugated circ_0008255 probe or scrambled control oligo probe, respectively. RNA bound to bead-probe complexes was subsequently purified using a RNeasy Mini Kit, and the enrichment levels of circ_0008255 and miR-192-5p were quantified via qRT-PCR.

**1.19. Characterization**

Transmission electron microscope (TEM, JEOL JEM-2100200 kV), scanning electron microscope (SEM, Hitachi Regulus 8100), X-ray crystal diffractometer (XRD, Rigaku Ultima IV X-ray diffractometer), dynamic light scattering (DLS, Nano ZS90), X-ray photoelectron spectroscopy (XPS, PHI Genesis 500), Fourier transform infrared spectrometer (FT-IR, Nicolet 6700), Fluorescence spectrometer (FLS 1000), Inductively coupled plasma emission spectrometer (ICP-OES, Agilent Technology 5100 Series), pH meter (Mettler Toledo FE28 Micro).

**1.20. Synthesis of Mg/Al LDH nanosheets**

Firstly, 3 mmol of Mg (NO_3_)_2_·6H_2_O and 1 mmol of Al (NO_3_)_3_·9H_2_O were dissolved in 3 mL of deionized water and slowly added dropwise to 40 mL of NaOH solution (225×10^-3^ M), and stirred at room temperature for 20 min. Then, the solution was centrifuged at a rate of 13000 rpm/min for 10 minutes to obtain a precipitate, which was washed 2-3 times with deionized water. The precipitate was sonicated again and dispersed in 40 mL of deionized water, and transferred to a high-pressure reaction vessel with a volume of 100 mL. The reaction was carried out at 100 ℃ for 12 hours. Finally, the reaction vessel was naturally cooled to room temperature, and the solution was centrifuged at a rate of 13000 rpm/min for 10 minutes to obtain a precipitate. The precipitate was washed three times by centrifugation with deionized water and ethanol, and freeze-dried to obtain Mg/Al LDH nanosheet powder.

**1.21. Synthesis of Mg/ Al-LDH@siRNA nanosheets**

Firstly, 10 mg Mg/Al LDH nanosheet powder was ultrasonically dispersed in 2 mL of deionized water. Then, siRNA was dissolved in 1 mL of deionized water and added to the nanosheet solution, which was stirred at room temperature for 12 hours. Subsequently, the solution was centrifuged at a rate of 13000 rpm/min for 10 minutes to obtain a precipitate. The precipitate was washed 2-3 times with deionized water to obtain Mg/ Al-LDH@siRNA nanosheets.

**1.22. Synthesis of Mg/ Al-LDH@siRNA@MM (LSM) nanosheets**

For cell membrane isolation: MM cells were harvested, suspended in hypotonic lysis buffer (20 mM Tris-HCl pH 7.5, 10 mM KCl, 2 mM MgCl₂, 1 EDTA-free mini protease inhibitor tablet/10 mL), and disrupted via 20 passes with a dounce homogenizer. After centrifugation at 3000×g for 5 min, the supernatant was collected; the pellet was resuspended in the same buffer to repeat the process. Supernatants from both rounds were pooled, centrifuged at 20,000×g for 20 min, then the resulting supernatant was centrifuged again at 100,000×g for 1.5 h. The final pellet was washed with 10 mM Tris-HCl (pH 7.5) + 1 mM EDTA, and the purified MM cell membrane was collected for subsequent experiments.

To prepare MM vesicles (MMs): The MM cell membrane was extruded through a 400 nm polycarbonate membrane for 11 passes. To prepare LSM nanosheets: LDH@siRNA nanosheets and MMs were mixed, then sequentially extruded through 400 nm and 200 nm polycarbonate membranes.

For both in vitro and in vivo experiments, the dosages of free LDH, LDH@siRNA and LSM groups were normalized based on equivalent LDH mass. The working concentration of LDH was set as 100 μg/mL for cellular assays, while the in vivo intravenous dosage was 20 mg/kg ^4^.

**1.23. Performance evaluation of LSM nanosheets releasing siRNA under different pH conditions**

Cy3 fluorescent dye was attached to siRNA (Cy3-siRNA) and loaded into LDH nanosheets for detecting the release performance of siRNA. Firstly, 10 mg LSM nanosheets were sonicated and dispersed in 50 mL of buffer solutions with different pH values (pH=7.4 and 4.5). Then, 1 mL of the solution was taken at different time points and centrifuged at a rate of 13000 rpm/min for 10 minutes. The supernatant was collected and the fluorescence intensity (E_x_=550 nm, E_m_=570 nm) was detected. Based on the fluorescence standard curve of Cy3-siRNA, the siRNA release curves of LSM nanosheets under different pH conditions were obtained.

**1.24. Lysosome staining**

Lysosome staining was performed strictly in accordance with the operating instructions of the Beyotime Lyso-Tracker Green Kit (Beyotime, C1047S). The specific procedure is as follows: After cells were subjected to different treatments, the prepared Lyso-Tracker Green working solution was added to the culture system and incubated at 37°C for 30 minutes. Subsequently, the staining working solution was removed, and fresh cell culture medium was added. After staining, observation was carried out using confocal microscope.

**1.25. Quantitative measurement of absolute lysosomal pH**

LysoSensor Yellow/Blue DND-160 ratiometric probe was used to calculate absolute intralysosomal pH with nigericin calibration. Myeloma cells in 96-well plates were treated with saline, LDH, LDH@siRNA or equivalent-dose LSM. After staining with the probe at 37 °C for 30 min and washing, in-well dual fluorescence (Ex 380 nm; Em 440 nm/540 nm) was detected, and Yellow/Blue ratios were recorded. To generate a pH standard curve, stained cells were incubated with pH 4.0–7.0 high-K⁺ buffers containing 10 μM nigericin (10 min) to equalize lysosomal and buffer pH. Measured ratios were fitted to a linear curve, which converted sample ratios into absolute pH values. All assays were performed in three biological triplicates.

**1.26. Chou-Talalay combination index (CI) analysis for synergism evaluation**

Chou-Talalay median-effect analysis was used to quantify synergistic anti-proliferative interactions between LDH and circ_0008255-targeted siRNA. For single-agent assays, RPMI8226 cells were exposed to serially diluted free LDH (100000–1000 ng/mL) or transfected with gradient concentrations of siRNA (1240–12.4 ng/mL) matched to the LDH mass ratio. Cell viability inhibition rates were measured by CCK-8. For combinatorial treatment, LDH@siRNA nanosheets were prepared at a fixed LDH:siRNA mass ratio of 100:1.24 and serially diluted consistent with the LDH concentration gradient. All dose-inhibition data of single LDH, single siRNA and combined formulation were fitted in CalcuSyn software to obtain median-effect parameters (Dm, m, r). CI values at ED50, ED75 and ED90 were calculated; CI < 1, = 1, and > 1 denote synergism, additive effect and antagonism, respectively.

**1.27. Statistical analysis**

All statistical analyses were conducted using SPSS 17.0 software. Student's t-test or analysis of variance (ANOVA) was performed for statistical analysis. Chi-square test was employed to compare the correlation between circ_0008255 expression and clinical indicators in MM samples. Data are presented as mean ± standard deviation. A p-value of less than 0.05 was considered statistically significant.

**References**

1.	[The guidelines for the diagnosis and management of multiple myeloma in China (2017 revision)]. *Zhonghua Nei Ke Za Zhi*. 2017 Nov 1;56(11):866-870.

2.	Wu H LC, Yang Q XC, Du J SF*, et al.* MIR145-3ppromotes autophagy and enhances bortezomib sensitivity in multiple myeloma by targetingHDAC4. *Autophagy*. 2019 .

3.	Luo, Z, et al. Inhibition of iRhom1 by CD44-targeting nanocarrier for improved cancer immunochemotherapy. Nat Commun 2024;15:255.

4.	Zhang, L, et al. Efficient Immunotherapy of Drug-Free Layered Double Hydroxide Nanoparticles via Neutralizing Excess Acid and Blocking Tumor Cell Autophagy. ACS Nano 2022;16:12036-12048.

**2. Supporting Figures**

**Fig. S1.** **A)** Length and abundance of circRNAs detected in RNA-seq. **B)** Genomic localization of circRNAs detected in RNA-seq. **C)** Volcano plot visualization of RNA-seq results. **D-F)** ROC curve analysis of circ_0008255 as a diagnostic indicator for MM. All the data are presented as the mean ± standard deviation from 3 independent experiments) n.s. *P* *>*0.05, * *P <*0.05, ** *P* < 0.01, *** *P <*0.001.

**Fig. S2.** **A)** Western blot was performed to detect the protein expression level of ABCC1 after circ_0008255 knockdown. **B)** In vivo imaging of tumor-bearing mice.

**Fig. S3.** **A)** Heatmap o**f** the expression level of autophagy-related genes from 50 normal and 413 multiple myeloma patients selected from GEO database. **B)** Western blot detection of LC3B and SQSTM1 after ATG2A knockdown. **C)** Knockdown of circ_0008255 in MM cells, stimulation with 3-MA (50 mM), western blot analysis of LC3B and SQSTM1 expression. **D)** Knockdown of circ_0008255 in MM cells, stimulation with rapamycin (50 nM), western blot analysis of LC3B and SQSTM1 expression. All the data are presented as the mean ± standard deviation from 3 independent experiments.

**Fig. S4.** **A)** RNA pull-down assay was performed to verify the direct interaction between circ_0008255 and miR-192-5p. **B)** RNA-FISH verified the interaction of circ_0008255 and miR-192-5p in U266 cells. All the data are presented as the mean ± standard deviation from 3 independent experiments, * *P <*0.05, ** *P* < 0.01, *** *P* <0.001.

**Fig. S5. A)** RT-qPCR detection of the overexpression efficiency of miR-192-5p in MM cells. **B)** CCK-8 assay for cell proliferation in MM cells overexpressing miR-ctrl or miR-192-5p. **C, D)** EdU assay for cell proliferation in MM cells overexpressing miR-ctrl or miR-192-5p (Scale bar = 500 μm). **E)** Colony formation assay for cell proliferation in MM cells overexpressing miR-ctrl or miR-192-5p (Scale bar = 500 μm). **F)** Western blot analysis of LC3B and SQSTM1 expression in MM cells overexpressing miR-ctrl or miR-192-5p. **G)** Western blot analysis of LC3B and SQSTM1 expression with ctrl or miR-192-5p inhibitor in MM cells. **H)** Observation of fluorescence in MM cells stably overexpressing LC3B and miR-192-5p treated with BAF (20 nM) (Scale bar = 25 μm). All the data are presented as the mean ± standard deviation from 3 independent experiments, * *P <*0.05, ** *P* < 0.01, *** *P <*0.001.

**Fig. S6.** Analysis results of fluorescence intensity in Fig. 5M. All the data are presented as the mean ± standard deviation from 3 independent experiments, * *P* <0.05, ** *P* < 0.01, *** *P* <0.001.

**Fig. S7.** Detection of ATG2A mRNA level changes upon simultaneous or separate overexpression of circ_0008255 and miR-192-5p in MM cells. All the data are presented as the mean ± standard deviation from 3 independent experiments, n.s. *P* >0.05, * *P* <0.05, ** *P* < 0.01, *** *P* <0.001.

**Fig. S8. A)** Volcano plot analysis based on RNA-seq results of overexpression of miR-192-5p in RPMI8226 cells. **B)** Detection of ATG2A mRNA level changes upon simultaneous miR-192-5p in MM cells. **C)** RT-qPCR assays detect the effects of knockdown efficiency of ATG2A. **D)** Western blot detection of ATG2A, LC3B and SQSTM1 after ATG2A knockdown. **E)** Confocal observation of fluorescence changes in MM cells expressing GFP-LC3B after ATG2A knockdown and treatment with BAF for 4 hours (Scale bar = 25 μm). **F)** CCK-8 assay for cell proliferation in MM cells after ATG2A knockdown. **G, H, I)** EdU and colony formation assay for cell proliferation in MM cells after ATG2A knockdown (Scale bar = 500 μm). **J)** Knockdown of circ_0008255 and ATG2A either simultaneously or separately in MM cells, followed by detection of ATG2A, LC3B and SQSTM1 expression. **K)** Overexpression of miR-192-5p and ATG2A either simultaneously or separately in MM cells with addition of BAF, followed by detection of ATG2A, LC3B and SQSTM1 expression. All the data are presented as the mean ± standard deviation from 3 independent experiments, * *P <*0.05, ** *P* < 0.01, *** *P <*0.001.

**Fig. S9.** Analysis results of fluorescence intensity in Fig. 6K. All the data are presented as the mean ± standard deviation from 3 independent experiments, * *P* <0.05, ** *P* < 0.01, *** *P* <0.001.

**Fig. S10.** Ovexpression of circ_0008255 and knockdown of ATG2A either simultaneously or separately in MM cells, followed by detection of ATG2A, LC3B and SQSTM1 expression.

**Fig. S11.** A) The CCK-8 assay was used to measure cell viability in different groups. B) Tumors were measured with an electronic caliper on days 5/7/9/11/13/15/17/19 in nude mice followed by statistical analysis. C) Weighing analysis of the tumors. D) The excised tumor from all the mice in each group. Comparison of tumor volume on day 19 after tumor removal. E) Live imaging of nude mice on day 19. All the data are presented as the mean ± standard deviation from 3 independent experiments, n.s. *P* >0.05, * *P* <0.05, ** *P* < 0.01, *** *P* <0.001.

**Fig. S12.** Characterization of material structure and composition. **A)** High magnification TEM image. **B)** The TEM image of Mg/Al LDH nanosheets. **C)** TEM images of cell membrane-coated LDH nanosheets. **D)** High resolution TEM. **E)** Selected area electron diffraction. **F)** XRD. **G)** Element Mapping. **H)** XPS. **I)** High resolution XPS spectra of Mg1s. **J)** High resolution XPS spectra of Al2p. **K)** High resolution XPS spectra of P2p. **L)** FTIR. **M)** The zeta potential of the LDH@siRNA nanosheets. **N)** UV-Vis absorption spectra of siRNA at different concentrations; **O)** Standard curve of siRNA (based on the absorbance at the characteristic absorption peak at 260 nm); **P)** UV-Vis absorption spectra of the supernatant obtained by centrifugation before and after adding LDH. **Q)** The fluorescence standard curve of Cy3-siRNA.

**Fig. S13.** **Antitumor effects of LSM in MM cells in vitro. A)** Intracellular delivery of LDH@siRNA nanosheets and LSM nanosheets on H929 cells after 4 h incubation observed by CLSM. **B)** Western blot assays were performed to characterize the expression of CD44, CD147 and CXCR4 across RPMI8226, U266 and MHCC97H cell lines. **C, D)** Intracellular delivery of LSM nanosheets on RPMI8226 cells after 4 h incubation observed by CLSM (Scale bar = 100 μm). **E)** CCK-8 assays were performed to detect the cell viability after treated with LSM. **F)** RT-qPCR assays were performed to detect the expression of circ_0008255 in H929 with different treatment. **G)** Cell proliferation changes in RPMI8226 measured by CCK-8 assay. **H, I)** Cell proliferation changes in H929 assessed by EdU staining. **J)** Western blot assay was performed to detect the expression of ATG2A, LC3B and SQSTM1. **K, L)** Confocal microscopy observation of cell autophagy in H929 stably expressing GFP-LC3B. **M, N)** Lysosomal staining is performed using lysosomal probes in H929 with different treatment (GFP: Lysosomal; Cy3: LDH@siRNA). **O)** The ratiometric fluorescence signals from LysoSensor Yellow/Blue DND-160 were measured via a microplate reader to calculate intracellular lysosomal pH in each treatment group. All the data are presented as the mean ± standard deviation from 3 independent experiments, n.s. *P* >0.05, * *P* <0.05, ** *P* < 0.01, *** *P <*0.001.

**Fig. S14. A)** Fa-CI plot. All experimental CI values below CI=1 threshold confirm synergistic effect. **B)** Median-effect plot. Linear fitting validates reliable dose-effect regression. **C)** Dose-effect curve. Concentration-dependent cell inhibition of LDH+siRNA.

**Fig. S15. In vivo antitumor efficacy and inhibitory effects of LSM on bone destructive lesions. A)** Ex vivo fluorescence imaging analysis of liver, spleen and lung from NOG mice at 48 hours after injection. **B)** Statistical graph of bioluminescence intensity in the femur, liver, spleen, and lung. **C)** Bioluminescence images of mice treated with saline (control, ctrl), LDH, LDH@siRNA, and LSM. **D)** Flow cytometry was used to quantify the percentage of CD138-positive cells in each group. **E)** Key pharmacokinetic parameters (AUC₀₋₁₂, t₁/₂, plasma CL) of three siRNA formulations. **F)** HE staining of femur from NOG mice with different treatments (Yellow scale bar = 500 μm. Black scale bar = 100 μm). **G)** TRAP staining of fermur from NOG mice with different treatments (Yellow scale bar = 500 μm. Black scale bar = 100 μm). n.s. *P* *>*0.05, * *P <*0.05, ** *P* < 0.01, *** *P <*0.001.

**Fig. S16.** **A)** HE staining was performed on the major organs (heart, liver, spleen, lung, and kidney) of mice in each treatment group (Scale bar = 100 μm). **B-E)** Hematological parameters of mice in each group were detected 7 days after injection, including lymphocyte counts (B), hematocrit (C), neutrophil counts (D), and platelet counts (E). **F-J)** Blood biochemistry analysis. Statistical data are presented as mean ± standard deviation (SD) (n = 6).

**Fig. S17** Immunohistochemical detection of LC3B expression in heart, liver, spleen, lung and kidney. (Scale bar = 100 μm)
