# Supplementary material for: LDH-mediated autophagic full-chain blockade for Multiple Myeloma treatment by targeting circ_0008255/miR-192-5p/ATG2A axis
Source: Bioact Mater. 2026 Aug 4;67:127–42. doi: 10.1016/j.bioactmat.2026.07.054 (PMC13471063; doi:10.1016/j.bioactmat.2026.07.054)
Supplement: Supplementary file 2 [file mmc2.docx]

**Table S1 Clinical features of 61 newly diagnosed MM patients.**

| Clinical features | No. of Cases (%) |
| --- | --- |
| Gender  Male  Female | 29 (47.5)  32 (52.5) |
| Age (Years)  < 65  ≥ 65 | 32 (52.5)  29 (47.5) |
| ISS Stage  Stage I  Stage II  Stage III | 10 (16.4)  17 (27.9)  34 (55.7) |
| M component at diagnosis  IgG type  IgA type  IgD type  Light chain type  Non secretory type | 28 (45.9)  12 (19.7)  4 (6.5)  15 (24.6)  2 (3.3) |

Abbreviations: ISS, International Staging System.

**Table S2 Clinical and pathological characteristics of 61 newly diagnosed MM patients.**

| Clinical parameters | Mean±SD |
| --- | --- |
| Albumin (g/L) | 35.08±4.85 |
| Hemoglobin (g/L) | 87.66±23.53 |
| β_2_M (mg/L) | 9.36±11.48 |
| Serum creatinine (μmol/L) | 141.75±178.14 |
| Calcium (mmol/L) | 2.29±0.25 |
| LDH (U/L) | 241.23±121.20 |
| IgG (g/L) | 28.77±29.09 |
| IgA (g/L) | 8.88±17.53 |
| IgM (g/L) | 0.28±0.20 |
| Serum kappa (g/L) | 5.21±6.85 |
| Serum lambda (g/L) | 4.63±6.52 |
| M protein (g/L) | 22.12±21.41 |

Abbreviations: β_2_M, beta-2-microglobulin; LDH, lactate dehydrogenase.

**Table S3 The summary of the ROC analysis data for circ_0008255, ALB, and HB as diagnostic markers for MM.**

| Test Items | AUC | 95%CI | Sensitivity | Specificity | Pvalue |
| --- | --- | --- | --- | --- | --- |
| circ_0008255 | 0.698 | 0.605-0.790 | 43.1 | 97.4 | <0.001 |
| ALB | 0.925 | 0.880-0.969 | 90.9 | 84.5 | <0.001 |
| HB | 0.887 | 0.818-0.956 | 98.7 | 75.9 | <0.001 |
| circ_0008255+ALB | 0.940 | 0.900-0.981 | 87.9 | 92.2 | <0.001 |
| circ_0008255+HB | 0.895 | 0.828-0.963 | 84.5 | 87.0 | <0.001 |
| circ_0008255+ALB+HB | 0.964 | 0.930-0.998 | 89.7 | 97.4 | <0.001 |

Abbreviations: ALB, albumin; HB, hemoglobin.

**Table S4 Chou-Talalay CI analysis of LDH combined with siRNA (100:1.24).**

| Summary table | | | | | | |
| --- | --- | --- | --- | --- | --- | --- |
| Drug | CI Values at | | |  | | |
|  | ED50 | ED75 | ED90 | Dm | m | r |
| LDH (Not a combination) | N/A | N/A | N/A | 15987 | 0.72774 | 0.97817 |
|  | | | | | | |
| siRNA (Not a combination) | N/A | N/A | N/A | 646.9 | 0.69013 | 0.97087 |
|  | | | | | | |
| LDH@siRNA (100:1.24) | 0.32444 | 0.43265 | 0.57762 | 3970 | 0.60492 | 0.97127 |

Abbreviations: LDH, layered double hydroxide.

**Table S5 Primer sequences**

| Name | Forward | Reverse |
| --- | --- | --- |
| hGAPDH | 5′-GCACCGTCAAGCTGAGAAC-3′ | 5′-TGGTGAAGACGCCAGTGGA-3′ |
| h-circ_0008255 | 5′-CTCAGCCTCTGGACTGATGAC-3′ | 5′-GAGGAGCTGCTGAGCTGTCTTG-3′ |
| h-circ_0027616 | 5′-AGAACCTGACATTCAAGACTCC-3′ | 5′-GCCAAACCTCCCTCCATAATG-3′ |
| h-circ_0009076 | 5′-GCAGTTCTTCATCCACCCAC-3′ | 5′-AGGCATCAAATCCATTCTCATCT-3′ |
| h-circ_0004939 | 5′-GGCTCCTGTTCCCTGTGAG-3′ | 5′-AGGGATGTGTGGGTGCTAC-3′ |
| ATG2A | 5′-CCTCTGTGAGACCAAGGATGAG-3′ | 5′-CCAGACAGAAGTAGCCAAGTCC-3′ |
| ABCC1 | 5′-CAACGGGACTCAGGAGCACA-3′ | 5′-CGGCCATGGAGTAGCCAAAC-3′ |
| miR-192-5p | 5′- GCGCGCTGACCTATGAATTG-3′ | 5′-TTATGGAGCCTGGGACGTGA-3′ |
| U6 | 5ʹ-GCTTCGGCAGCACATATACTAAAAT-3 | 5ʹ-CGCTTCACGAATTTGCGTGTCAT-3’ |
| circ_0008255 siRNA | 5’-GGCGGCATTTCACAAGACA-3’ | 5’-TATCCTTCTTCACGACTCCTTCAC-3’ |

**Table S6 shRNA sequences**

| Gene | shRNA sequence |
| --- | --- |
| h­_circ_0008255F | 5’-CcggGGCATTTCACAAGACAGCTCACTCGAGTGAGCTGTCTTGTGA  AATGCCTTTTTTg-3’ |
| h­_circ_0008255R | 5’-aattcaaaaaaGGCATTTCACAAGACAGCTCACTCGAGTGAGCTGTCTT  GTGAAATGCC-3’ |
| h­_ATG2A_F | 5’-CcgggaTGTTGACCTCTTGGAACTTCTCGAGAAGTTCCAAGAGGTCA  ACATCTTTTTg-3’ |
| h­_ATG2A_R | 5’-aattcaaaaagaTGTTGACCTCTTGGAACTTCTCGAGAAGTTCCAAGAG  GTCAACATC-3’ |
